# Genome-Wide Identification, Evolution, and Expression Analysis of GASA Gene Family in *Prunus mume*

**DOI:** 10.3390/ijms231810923

**Published:** 2022-09-18

**Authors:** Man Zhang, Wenhui Cheng, Jia Wang, Tangren Cheng, Qixiang Zhang

**Affiliations:** Beijing Key Laboratory of Ornamental Plants Germplasm Innovation & Molecular Breeding, National Engineering Research Center for Floriculture, Beijing Laboratory of Urban and Rural Ecological Environment, Engineering Research Center of Landscape Environment of Ministry of Education, Key Laboratory of Genetics and Breeding in Forest Trees and Ornamental Plants of Ministry of Education, School of Landscape Architecture, Beijing Forestry University, Beijing 100083, China

**Keywords:** GASA gene family, *Prunus mume*, evolutionary analysis, gene family analysis, gibberellin-responsive genes

## Abstract

The Gibberellic Acid Stimulated Arabidopsis/Gibberellin Stimulated Transcript (GASA/GAST) gene family is a group of plant-specific genes encoding cysteine-rich peptides essential to plant growth, development, and stress responses. Although GASA family genes have been identified in various plant species, their functional roles in *Prunus mume* are still unknown. In this study, a total of 16 *PmGASA* genes were identified via a genome-wide scan in *Prunus mume* and were grouped into three major gene clades based on the phylogenetic tree. All PmGASA proteins possessed the conserved GASA domain, consisting of 12-cysteine residues, but varied slightly in protein physiochemical properties and motif composition. With evolutionary analysis, we observed that duplications and purifying selection are major forces driving *PmGASA* family gene evolution. By analyzing *PmGASA* promoters, we detected a number of hormonal-response related cis-elements and constructed a putative transcriptional regulatory network for *PmGASAs*. To further understand the functional role of *PmGASA* genes, we analyzed the expression patterns of *PmGASAs* across different organs and during various biological processes. The expression analysis revealed the functional implication of *PmGASA* gene members in gibberellic acid-, abscisic acid-, and auxin-signaling, and during the progression of floral bud break in *P. mume*. To summarize, these findings provide a comprehensive understanding of GASA family genes in *P. mume* and offer a theoretical basis for future research on the functional characterization of GASA genes in other woody perennials.

## 1. Introduction

The Gibberellic Acid Stimulated Arabidopsis/Gibberellin Stimulated Transcript (GASA/GAST) is a plant-specific gene family widely present in gymnosperms, angiosperms, and pteridophytes [1]. GASA family genes encode cysteine-rich peptides (CRPs) consisting of a secretory peptide signal at the N-terminal, a highly hydrophilic middle part, and the GASA domain at the C-terminal [2]. The GASA domain is a conserved 60 amino acid domain containing 12 featured cysteine residues arranged in a repetitive pattern [3,4]. In previous studies, proteins lacking a complete GASA domain were found to be non-functional [5]. GASA family proteins were first reported in tomato (*Lycopersicon esculentum*) [6] but, over the past decades, have then characterized in many other plant species, including *Arabidopsis* [7], rice (*Oryza sativa*) [8], potato (*Solanum tuberosum*) [9], and poplar (*Populus trichocarpa*) [10]. However, the GASA gene family was absent in charophytes or bryophytes [3,11].

GASA family genes play a vital role in plant development and reproduction [12]. In *Arabidopsis*, AtGASA4 is reported to regulate floral meristem identity and positively influence seed development [12]. AtGASA5, on the other hand, is involved in the gibberellin-promoted flowering in *Arabidopsis* [13]. The over-expression of *AtGASA5* leads to delayed flowering time through enhancing the transcription of the flowering repressor gene, *FLC* (FLOWERING LOCUS C) and repressing *FT* (FLOWERING LOCUS T) and *LFY* (LEAFY) [13]. *AtGASA10* encodes a cell wall protein that functions in promoting cell elongation in developing anthers and seeds [14]. FaGAST1 and FaGAST2, two GAST-like proteins isolated from strawberry, act together to determine cell size during fruit development and ripening [15]. In *Gerbera hybrida*, *GhGEG*, the Gerbera ortholog of tomato *GAST1*, is reported to inhibit cell elongation during ray petal development [16,17]. For perennial trees, GASA gene members are reported to potentially regulate floral induction in apple (*Malus domestica*) [18] and bud dormancy cycling in pear (*Pyrus pyrifolia*) [19].

GASA family genes are also involved in hormone signaling transduction and biotic/abiotic stress tolerance [10,20]. Most GASA genes are part of the gibberellin pathways. For example, six out of fifteen GASA genes (*AtGASA1*, *AtGASA4*, *AtGASA6*, *AtGASA7*, *AtGASA8*, and *AtGASA13*) are responsive to the exogeneous application of gibberellin in *Arabidopsis* [21]. In *Glycine max*, *GmGASA32* is up-regulated by gibberellin and promotes plant height by interacting with GmCDC25 (cell cycle-associated protein) [22]. GASA family genes are also responsive to other phytohormones. In rice, OsGSR1, a GASA family gene, mediates the crosstalk between brassinosteroid (BR) and GA signaling pathways [2]. Acting as the integrator of gibberellin, abscisic acid (ABA), and glucose signaling, *AtGASA6* regulate seed germination and hypocotyl elongation through connecting the *AtRGL2* (RGA-LIKE 2) and *AtEXPA1* (EXPANSIN A1) gene in *Arabidopsis* [23]. The constitutive expression of *AtGASA14* in transgenic *Arabidopsis* exhibited an elevated Reactive Oxygen Species (ROS) level and increased tolerance to salt stress [24]. In addition, GASA family proteins are linked with resistance to bacterial and fungal pathogens. In *Hevea brasiliensis*, HbGASA genes are also found to be involved in regulating innate immunity through modulating ROS accumulation to repressing fungal pathogens [25].

*Prunus mume*, commonly known as Japanese apricot, is an important ornamental and fruit tree species widely cultivated in East Asia [26]. The fruits of *Prunus mume* can be processed into preserved delicacies and wine, or applied for medical purposes [27]. *Prunus mume* is a deciduous tree species with more than 1500 years cultivation history and is highly prized for its delicate blossoms and pleasant fragrance [28]. Therefore, *Prunus mume* is also considered as an asset to landscape and gardening [29]. GASA family genes are implicated in many important plant biological processes. However, their functional roles are still uncharacterized in *Prunus mume*. In this study, we identified GASA family genes and analyzed their gene structure, phylogenetic relationships, protein chemical properties, and conserved motif, as well as their putative regulatory networks in *Prunus mume*. With genome synteny and selection analysis, we clarified the evolutionary trajectory of GASA family genes in *Prunus mume*. Furthermore, we performed spatial and temporal expression analysis to investigate the functional role of GASA family genes during floral development and hormonal response processes in *Prunus mume*. Our findings provide comprehensive insights into the evolution and functional importance of GASA family genes in *Prunus mume*.

## 2. Results

### 2.1. Genome-Wide Identification of GASA Genes across Six Plant Species

Previous studies reported a total of fifteen *GASA* genes in *A. thaliana* [21]. To identify GASA family genes in other plant species, we employed two approaches, combining Hmmer and BLASTP search. With genome-wide scans, we identified a total of 11 GASA genes for Asian rice (*Oryza sativa*), 28 from apple (*Malus domestica*), 16 from *Prunus mume*, 19 from poplar (*Populus tricocarpa*), and 17 from peach (*Prunus persica*) (Table 1). All 106 GASA family genes were manually verified using conserved domain search tools (https://www.ncbi.nlm.nih.gov/Structure/cdd/wrpsb.cgi; accessed on 24 June 2022) and the Pfam database containing GASA domains (Table 1 and Table S2). The GASA family proteins were further blasted against the NCBI non-redundant protein sequence database, with their closest gene accession returned (Table 1 and Table S2). The GASA gene family members from each species are renamed by their sequential order across chromosomes. All detailed information of the *GASA* genes analyzed in this study were listed (Table 1 and Table S2).

### 2.2. Gene Structure and Protein Motif Analysis of PmGASAs

**Figure 1:**
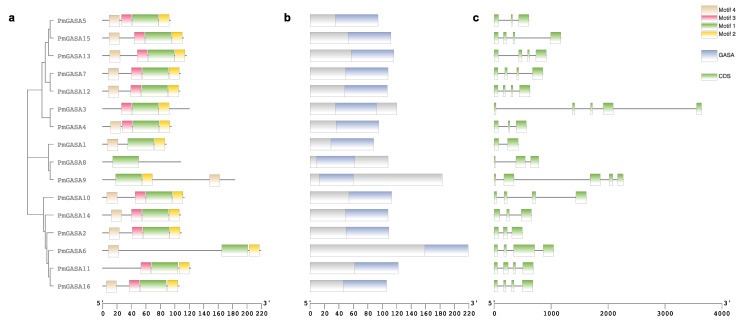
Protein motif and gene structure analysis of GASA family genes identified in *P. mume*. (**a**) Phylogenetic tree of PmGASA protein sequences with conserved protein motifs colored differently. (**b**) Conserved protein domain identified for *PmGASA* proteins using the NCBI CDD tool. The blue box indicates the conserved GASA domain identified. (**c**) Exon–intron distribution analysis of *PmGASA* genes. The green boxes represent exons and the black lines represent intron positions, respectively.

The GASA family genes were distributed unevenly across eight chromosomes in *P. mume* (Figure S1). Out of 16 genes, four (*PmGASA1–4*) were mapped to chromosome 2 (Figure S1). Gene clusters *PmGASA5–7* and *PmGASA8–10* were mapped to chromosome 4 and chromosome 6, respectively (Figure S1). *PmGASA11–12* and *PmGASA13–14* were located to chromosome 7 and chromosome 8, respectively, and the other two genes were present in the form of scaffolds (Figure S1). All PmGASA proteins contained the GASA domain and can be grouped into three sub-clusters based on the polygenetic tree (Figure 1). Protein motif analysis revealed four featured motifs, including RRCSLTSRKKPCMRFCGKCCEKCLCVPPGTYGNKEEC (motif 1), PCYNNWKTKGGGPKC (motif 2), GPGSLRPIECGSACT (motif 3), and FLLLALLLLSMVAEV (motif 4) (Figure S2). Among the four motifs, motif 1 was universally present on every member of the PmGASA family proteins (Figure 1a). PmGASA3, PmGASA8, and PmGASA11 lack protein motif 4, while only PmGASA8 lacks motif 2 (Figure 1a).

**Figure 2:**
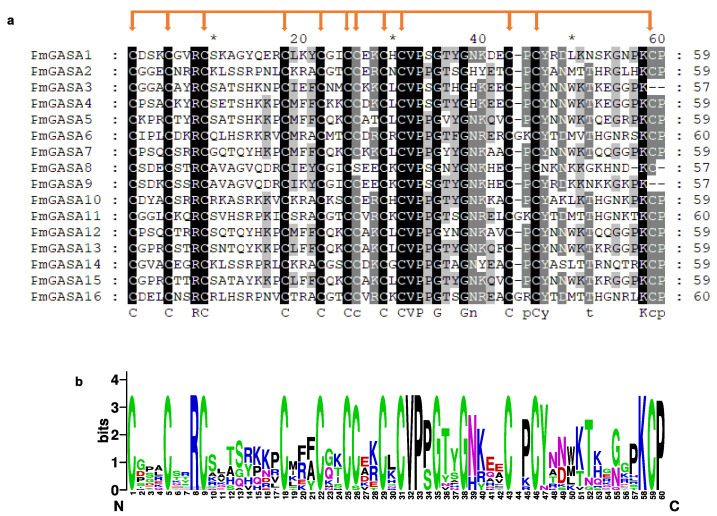
Amino acid alignment of GASA domain from PmGASA proteins. (**a**) Multiple alignment of the PmGASA protein sequences within the GASA domain. The alignment was visualized in GeneDoc, with grey shading indicating the amino acid identity. The featured cysteines within the GASA domain are highlighted with red arrows. The 10^th^, 30^th^, 50^th^ amino acid residues were labeled with *. (**b**) Sequence logo analysis of the conserved GASA domains performed with WebLogo platform.

The exon/intron distribution of *PmGASA* genes were analyzed based on the gene annotation of the *P. mume* genome (Figure 1c). The exon number of *PmGASA* genes ranged from two to five. The largest numbers of exons were present in *PmGASA3* and *PmGASA9* (five exons), while *PmGASA1* contained only two exons. The exon/intron size was generally conserved among *PmGASA* genes, except for *PmGASA3*, *PmGASA9*, *PmGASA10*, and *PmGASA15*, with relatively long introns of more than 500 bp (Figure 1c). To further examine the conservation of the GASA domain, we extracted the GASA domain sequence of PmGASA proteins and performed multiple alignment. All PmGASA proteins were shown to possess the 12 conserved cysteines within the GASA domain region, except for PmGASA3 and PmGASA9, which both lacked the 12th featured cysteine, which is likely attributed to amino acid mutations (Figure 2).

### 2.3. Physiochemical Analysis of PmGASA Proteins

Protein sequences of PmGASAs ranged from 88 to 219 amino acids, with a molecular weight between 9613.29 and 23,383.3 Da (Table 2). By assessing the physiochemical properties of PmGASA proteins, we determined the isoelectric point (pI), instability index, and the grand average of hydropathy (GRAVY) using the ExPASy tool. Most PmGASA family proteins shared relatively high pI values, among which fifteen had pI value > 8.0 (Table 2). The instability index of most PmGASA proteins are higher than 40, except for PmGASA1, PmGASA12, and PmGASA15 (Table 2). The aliphatic index of PmGASAs ranged from 28.67 to 87.72, which is approximately equivalent to that of GASA proteins in other plant species [3]. The GRAVY values of all PmGASAs are below −0.018, indicating that all PmGASA proteins are hydrophilic (Table 2). The subcellular localization of *PmGASA* genes was predicted to be in the cellular nucleus and golgiosome (Table 2). In the 3D structure model of PmGASA proteins, they are predicted to possess random coils. However, we observed no transmembrane helix on PmGASA proteins.

### 2.4. Phylogenetic Analysis of GASA Family Genes

**Figure 3:**
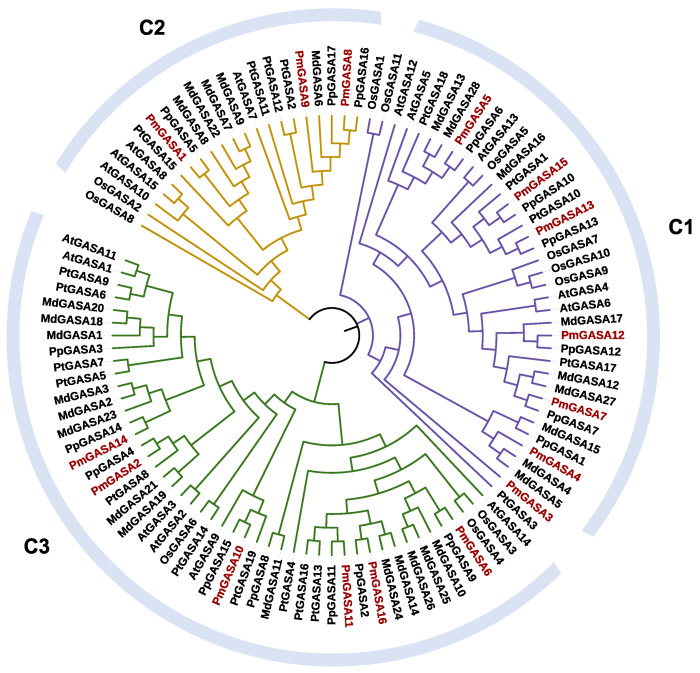
Phylogenetic analysis of 106 GASA family genes from six plant species based on their protein alignment. The phylogenetic tree was constructed using the NJ method, with 1000 bootstrap replicates in MEGA. The GASA genes were clustered into three clades (C1, C2, C3), each one colored differently.

To study the phylogeny of GASA family genes in *P. mume*, we constructed the phylogenetic tree for 106 GASA proteins from *A. thaliana*, *O. sativa*, *M. domestica*, *P. mume*, *P. persica*, and *Populus tricocarpa* (Figure 3). The GASA family genes can be divided into three major clades, with Clade 1 containing 38 genes, Clade 2 containing 21 genes, and Clade 3 containing 47 genes (Figure 3). All three clades have genes from six species. Among 16 *PmGASA* genes, *PmGASA3*, *PmGASA4*, *PmGASA5*, *PmGASA7*, *PmGASA12*, *PmGASA13*, and *PmGASA15* were clustered with *AtGASA4*, *AtGASA5*, *AtGASA6*, *AtGASA12*, *AtGASA13* into Clade 1 (Figure 3). Three *PmGASA* genes (*PmGASA1*, *PmGASA8*, and *PmGASA9*) fell into Clade 2 with *AtGASA7*, *AtGASA8*, *AtGASA10*, and *AtGASA15* (Figure 3). The remaining six *PmGASA* genes were clustered into Clade 3 with *Arabidopsis* orthologs *AtGASA1*, *AtGASA2*, *AtGASA3*, *AtGASA9*, *AtGASA11*, and *AtGASA14* (Figure 3). Within each subgroup, orthologous genes of *P. mume* were first clustered with *P. persica*, then with *M. domestica*, and with *Populus tricocarpa*, *Arabidopsis*, and finally with *O. sativa* (Figure 3). *OsGASA* proteins were clearly separated from their GASA orthologs in other dicotyledonous species (Figure 3). Among all species, the number of GASA family genes in *M. domestica* is the highest within each clade, which is almost twice that in *Arabidopsis* and in *P. mume*.

### 2.5. Evolutionary Analysis of GASA Family Genes in P. mume and Other Species

**Figure 4:**
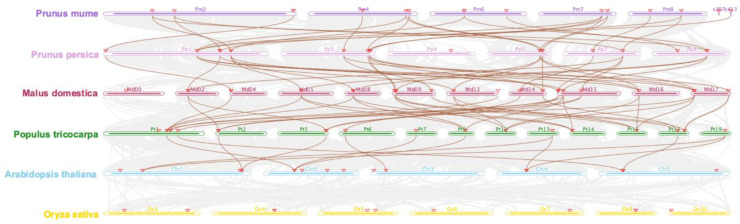
Synteny analysis of *GASA* genes between *Arabidopsis thaliana*, *Malus domestica*, *Prunus mume*, *Prunus persica*, *Populus tricocarpa*, and *Oryza sativa.* Red triangle boxes highlight the GASA family genes. The grey shade highlights syntenic regions between genomes, and the colored lines represent colinear *GASA* gene pairs.

To characterize the evolutionary mechanism of *GASA* family genes, we conducted all-against-all BLASTP and identified collinearity blocks across the genomes of six plant species. In general, we observed extensive genome synteny between *P. persica* and *P. mume*, *P. persica* and *M. domestica*, and *Populus tricocarpa* and *A.thaliana* (Figure 4). However, the synteny is less abundant between genomes of *A. thaliana* and *O. sativa*. In our study, we identified 13 *PmGASA* genes in collinearity with 13 *PpGASAs*, which are located within the syntenic regions with 18 orthologs from *M. domestica* (Figure 4). In *A. thaliana*, only seven *GASAs* have orthologous gene pairs in *Populus tricocarpa* (Figure 4). However, we detected no corresponding collinear *GASA* orthologs between *A. thaliana* and *O. sativa*.

We also analyzed the duplication events of GASA family genes among six species (Table S3). Dispersed duplications, tandem duplications, and WGD/segmental duplications are three gene duplication modes mainly involved in the evolution of the GASA family genes among dicotyledonous plant species (Table S3). Proximal duplications were only reported in *MdGASA26*, *MdGASA9*, and *PpGASA17* (Table S3). In *O. sativa*, dispersed and singleton duplications are two major types of duplications responsible for GASA gene family expansion (Table S3). In *P. mume*, a tandem duplication event should have generated the gene cluster containing *PmGASA3* and *PmGASA4* on chromosome 2 (Table S3). On the other hand, WGD or segmental duplications were detected across different chromosomes, generating four gene pairs including *PmGASA1*–*PmGASA8*, *PmGASA2*–*PmGASA14*, *PmGASA6*–*PmGASA11*, and *PmGASA7*–*PmGASA12* (Figure S3 and Table S3). KaKs tests were conducted on these five duplicated *PmGASA* gene pairs (Table 3). All five gene pairs have Ka/Ks ratios of less than 1, which suggests that the duplicated *PmGASA* gene pairs have undergone strong purifying selection in the course of evolution (Table 3). To understand the adaptive selection pressure on *PmGASA* proteins, we also performed selection tests on three gene clades separately using software Selecton. The Clade 1 and Clade 3 genes all showed strong signs of purifying selection among most amino acid sites, indicating the conserved protein evolution (Figure S4). In contrast, genes within *PmGASA* Clade 3 revealed a signature of positive selection on 41 residues, while the rest of the amino acids were under purifying selection (with *PmGASA9* as reference gene) (Figure S4).

### 2.6. Promoter Analysis of PmGASA Genes and Their Possible Activators

**Figure 5:**
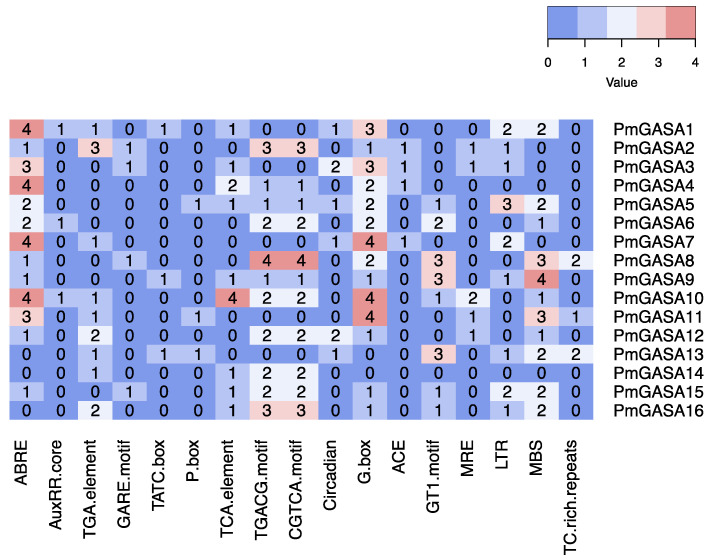
Analysis of cis-regulatory elements within *PmGASA* gene promoters. The numerical values and colors represent the number of cis-elements identified within each *PmGASA* gene.

We first analyzed the cis-regulatory elements within the promoter regions of *PmGASA* genes (Figure 5 and Table S4). We detected a number of hormone responsive elements. For example, the ABA (abscisic acid) responsive element ABRE, the auxin responsive elements (AuxRR-core and TGA-element), the gibberellin responsive elements (GARE-motif, TATC-box, and P-box), the salicylic acid (SA) responsive TCA-element, and the Methyl Jasmona (MeJA) responsive elements (TGACG-motif and CGTCA-motif) were identified (Figure 5 and Table S4). Among the *PmGASAs*, the gibberellin responsive elements were absent from *PmGASA4*, *PmGASA6*, *PmGASA7*, *PmGASA10*, *PmGASA12*, *PmGASA14*, and *PmGASA16* promoters (Figure 5). The ABRE element was abundantly detected within most *PmGASA* promoters, except for *PmGASA13*, *PmGASA14*, and *PmGASA16* (Figure 5). Moreover, we identified abiotic-stress related elements, such as light responsive cis-elements (G-box, ACE motif, GT1-motif, and MRE motif), LTR (low-temperature responsiveness), MBS (drought-inducibility), and TC-rich repeats (defense and stress responsiveness) (Figure 5 and Table S4).

We also inferred the possible transcriptional regulatory network of *PmGASAs* using the online tool PlantRegMap (Figure S5). The predicted transcription factor (TFs) acting upstream of *PmGASA* genes included the AREB (ABA-RESPONSIVE ELEMENT BINDING PROTEIN) transcription factors, the ARF (AUXIN RESPONSE FACTOR) family transcription factors, the bZIP (BASIC LEUCINE-ZIPPER) transcription factors, and the MYB family transcription factors (Figure S5). Among the TFs, the bZIP transcription factors, such as bZIP16, bZIP42, bZIP44, bZIP53, and bZIP63 are putative activators of *PmGASA12*. Additionally, a MADS-box transcription factor SOC1 (SUPPRESSOR OF OVEREXPRESSION OF CO 1) is predicted to target *PmGASA7*, *PmGASA9*, *PmGASA13*, *PmGASA14*, *PmGASA15*, and *PmGASA16* in *P. mume* (Figure S5). We also detected a few hormone responsive transcription factors. For example, RGA1 (REPRESSOR OF GA1), a transcriptional regulator acting on upstream *PmGASA1*, *PmGASA13*, *PmGASA14*, *PmGASA15*, and *PmGASA16* represses gibberellin responses (Figure S5). The ABA induced transcription factors, including ABF2 (ABSCISIC ACID RESPONSIVE ELEMENTS-BINDING FACTOR 2), ABI5 (ABA INSENSITIVE 5), and AREB3 (ABSCISIC ACID RESPONSIVE ELEMENTS-BINDING POTEIN 3), are predicted to bind the ABRE elements within the promoters of *PmGASA7* and *PmGASA12* (Figure S5). In addition, DOF (DNA BINDING WITH ONE FINGER), OBP (OBF-binding protein), and BPCs (BASIC PENTACYSTEINE) family TFs were also predicted to be the transcriptional activators for *PmGASA3*, *PmGASA7*, *PmGASA9*, *PmGASA13*, *PmGASA14*, and *PmGASA15*.

### 2.7. Tissue-Specific Expression Patterns of PmGASA Genes

**Figure 6:**
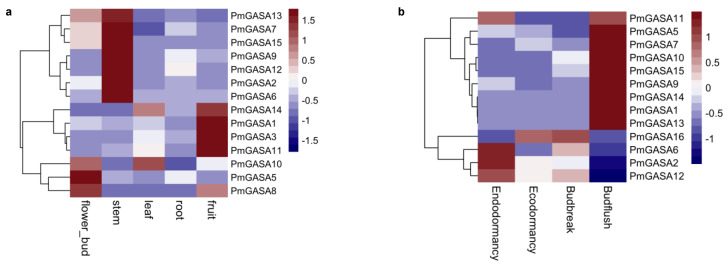
Expression analysis of *PmGASA* genes across different organs and during floral bud development in *P. mume*. (**a**) Expression profile of *PmGASAs* in flower bud, leaf, root, and fruit tissues. (**b**) Expression profile of *PmGASAs* during four developmental stages of floral bud break. Hierarchical clustering was used to compare the gene expression levels of *PmGASAs* across different samples, with highly expressed genes shown in red and weakly expressed in blue.

We investigated the expression patterns of *PmGASA* genes across different organ tissues of *P. mume* (Figure 6a). Among all genes, *PmGASA5*, *PmGASA8*, and *PmGASA10* were preferentially high-expressed in flower bud tissues (Figure 6a). *PmGASA1*, *PmGASA3*, *PmGASA11*, and *PmGASA14* were relatively high-expressed in fruits rather than other organs (Figure 6a). *PmGASA2*, *PmGASA6*, *PmGASA7*, *PmGASA9*, *PmGASA12*, *PmGASA13*, and *PmGASA15* were more high-expressed in stems (Figure 6a). *PmGASA7*, *PmGASA13*, and *PmGASA15* were also expressed in flower bud tissues (Figure 6a). All *PmGASA* genes were relatively low-expressed in root tissues (Figure 6a).

### 2.8. Expression Patterns of PmGASA Genes during Floral Bud Development

To understand the functional role of *PmGASA* genes in floral bud development, we analyzed the expression levels of *PmGASAs* during the flower bud break process (Figure 6b). The *PmGASA* genes can be clustered into three major groups based on their expression profiles. *PmGASA1*, *PmGASA5*, *PmGASA7*, *PmGASA9*, *PmGASA10*, *PmGASA11*, *PmGASA13*, *PmGASA14*, and *PmGASA15* were highly induced during the flower bud flushing stage (Figure 6b). The transcription levels of *PmGASA2*, *PmGASA6*, and *PmGASA12* were relatively high in the endodormant floral bud, and then significantly dropped after flower buds exited dormancy (Figure 6b). *PmGASA16* was strongly induced after endodormancy release, but was then found to be repressed in flushed flower buds (Figure 6b).

### 2.9. Expression Patterns of PmGASA Genes in Response to Gibberellin Treatments

**Figure 7:**
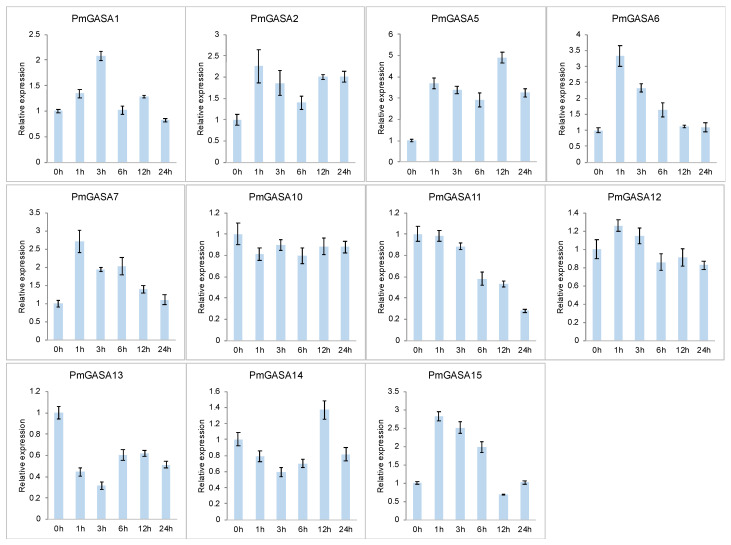
Relative expression levels of *PmGASAs* in leaves over two days, after gibberellin treatment. Error bars represent the standard deviations assessed from three technical replicates.

To further explore the function of *GASA* genes during gibberellin responses, we first investigated the expression patterns of *PmGASA* genes in leaves after hormone treatment (Figure 7). It was shown that *PmGASA1*, *PmGASA2*, *PmGASA5*, *PmGASA6*, *PmGASA7*, and *PmGASA15* were strongly induced within one hour after the GA treatment (Figure 7). Among them, *PmGASA6* and *PmGASA7* showed the highest up-regulation within one hour after GA treatment, approximately three-fold (Figure 7). On the contrary, we observed that *PmGASA11*, *PmGASA13*, and *PmGASA14* were firstly down-regulated within a short period of time in response to gibberellin treatment (Figure 7). The expression of *PmGASA11* maintained a constant level within the first three hours, but then dropped after six hours, while the transcript levels of *PmGASA13* and *PmGASA14* increased slightly 12 hours after gibberellin treatment (Figure 7).

To assess the effects of GA treatment on *PmGASA* genes in floral bud, we also analyzed the expression patterns of *PmGASA* in flower buds treated with GA_3_. The transcription of *PmGASA1*, *PmGASA2*, *PmGASA5*, *PmGASA6*, *PmGASA7*, and *PmGASA11* was highly induced in flower buds within three hours after GA treatment and was found slowly repressed after six hours (Figure S6). Meanwhile, the expression levels of *PmGASA10* and *PmGASA15* first dropped, but were slightly increased three hours after GA treatment (Figure S6). *PmGASA12* and *PmGASA13*, on the other hand, were repressed at all time after GA treatment in the flower buds of *P. mume* (Figure S6). However, the rest of the *PmGASA* genes, such as *PmGASA4*, *PmGASA9*, and *PmGASA16* were barely detected in the GA-treated leaves or floral buds.

### 2.10. Expression Patterns of PmGASA Genes in Response to ABA and IAA Treatments

**Figure 8:**
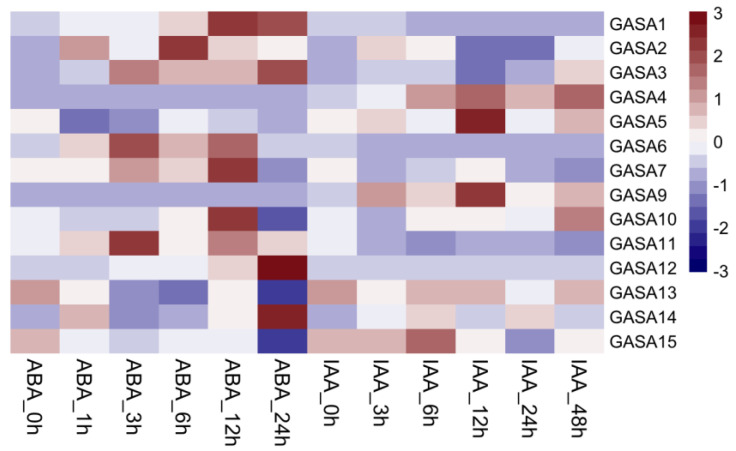
Expression analysis of *PmGASAs* in response to ABA and IAA treatments. The heatmap displayed the average relative expression levels of *PmGASAs* from the qRT-PCR assays.

To test the involvement of *PmGASA* genes in the signaling transduction of other phytohormones, we analyzed the expression profile of *GASA* genes in leaves treated with exogenous ABA and IAA (Figure 8). Upon the application of ABA, the expression levels of *PmGASA1*, *PmGASA2*, *PmGASA6*, *PmGASA11*, *PmGASA12*, and *PmGASA14* were instantly up-regulated, while *PmGASA5*, *PmGASA13*, and *PmGASA15* were down-regulated within a few hours and were then slightly increased (Figure 8). After the treatment of IAA, *PmGASA1* and *PmGASA2* were strongly induced within one hour, but *PmGASA4*, *PmGASA14*, and *PmGASA15* displayed slowly increasing expression patterns during the first 12 h (Figure 8). On the other hand, *PmGASA6*, *PmGASA11*, *PmGASA7*, and *PmGASA12* were down-regulated after IAA treatment (Figure 8). However, the expression levels of *PmGASA7* and *PmGASA12* were strongly increased approximately at 12 h after the IAA treatment (Figure 8).

## 3. Discussion

Gibberellins are a class of phytohormones involved in many important plant developmental processes. GASA/GAST is a multi-gene family regulated by gibberellin acid and encode small cysteine-rich peptides that are implicated in regulating plant growth and development [1]. The GASA family gene, *GAST1*, was firstly discovered in tomato (*Solanum lycopersicum*) as a GA-stimulated protein [6]. So far, GASA gene members have been identified in a wide range of plant species, such as *Arabidopsis thaliana* [21], soybean (*Glycine max*) [30], cotton (*Gossypium hirsutum*) [31,32], potato (*Solanum tuberosum*) [33], strawberry (*Fragaria* × *ananassa*) [15], rice (*Oryza sativa*) [2,8], and grapevine (*Vitis vinifera*) [34] etc. GASA family genes were reported to regulate seed germination [23], the development of lateral root [35], flower organ formation [16], fruit development [15], stem growth and flowering time [13], and environmental stress responses [24,36].

Despite previous studies of *GASA* family genes in different plant species, their functional roles in perennial tree species have not been fully uncovered. In poplar tree, *PeuGASAs* were found to be induced or inhibited by different hormones, including ABA, SA, and MeJA, and were responsive to drought, mechanical damage, and other abiotic stresses [10]. *MdGASA* genes were also found responsive to GA and ABA treatment and were potentially related to flower induction in apple (*Malus domestica*) [18]. In white pear (*Pyrus pyrifolia*), PpyGAST1 potentially regulates bud dormancy by integrating GA and ABA signaling [19]. To obtain a deeper understanding of the structure and function of GASA members in woody perennials, we comprehensively investigated the phylogenetic relationship, exon-intron structure, protein motif, and cis-element analysis of GASA family genes. We also analyzed the molecular evolution of GASA genes and explored their functional roles in *P. mume*, combining transcriptional regulatory network prediction and expression analysis.

In our study, we identified 16 GASA protein encoding genes in *P. mume* and 90 *GASA* genes from the other five species (*A. thaliana*, *M. domestica*, *P. mume*, *P. persica*, *Populus tricocarpa*, and *O. sativa*). The gene number of GASA gene family is approximately even among species, except *M. domestica*. We detected 28 *MdGASA* genes in *M. domestica*, approximately two-fold of that in *Prunus* species. This is likely due to a whole genome duplication event occurring in *Maleae* species after splitting from *Prunus* [37]. By examining the molecular structure of *PmGASAs*, we observed relatively consistent exon-intron placement and protein motifs among GASA genes, which is in compliment with that of GASA genes in other tree species [10,18]. The slight structural variation among GASA genes is likely due to the gain or loss of exons/introns during chromosomal evolution [30]. PmGASA proteins consist of 88 to 219 amino acids, which is similar to the AtGASA proteins of 80–275 amino acid residues [21]. The GASA domain within most PmGASA proteins possessed the featured cys-motif of twelve cysteine residues, which is essential to maintain the spatial structure and function of GASA proteins [20]. The cysteine residues are required for the formation of disulfide bonds, which are critical for GASA protein folding and interaction with other proteins [38,39]. The PmGASA proteins were predicted to be hydrophilic and mostly unstable, except for PmGASA1, PmGASA12, and PmGASA15. The subcellular localization of PmGASA proteins is in the nucleus or golgiosome. In previous studies, the GASA proteins were detected in the plasma membrane, cell wall, cytoplasm, nuclei, or extracellular space in different plant species [8,13,24]. Despite the presence of an N-terminal signal peptide, post-translational modifications, protein-protein interaction, and covalent bonds to lipids may be responsible for the proper localization of PmGASA proteins [8,13,24].

The phylogenetic analysis revealed that the GASA gene members of six plant species are clustered into three distinct clades, which is commonly observed in angiosperms [23]. The *PmGASAs* are grouped with GASA family members from *P. persica*, then with other dicotyledonous species, including *M. domestica*, *Populus tricocarpa* and *A. thaliana*, and finally with the monocotyledonous species *O. sativa*. The GASA gene phylogeny coincides with the species phylogenic tree [40]. To understand the evolutionary trajectory of *GASA* family genes, we inferred the gene duplication events of GASA family genes among five plant species. In dicotyledonous species, dispersed duplications, tandem duplications, and WGD or segmental duplications have played a dominant role during the GASA gene family evolution. Dispersed and singleton duplications, however, are two predominant duplication modes observed in monocotyledonous species. As for *P. mume*, the WGD/segmental duplications generated four gene pairs, while tandem duplications generated one gene pair. The Ka/Ks ratio between the paralogous genes of five *PmGASA* gene pairs were all less than 1, suggesting that the duplicated gene pairs are under strong purifying selection restraining their coding sequence variation. With protein selection analysis, we observed strong purifying selection acting on PmGASA proteins within Clade 1 and Clade 3 [30]. However, adaptive selection pressure was detected at a few specific residues for PmGASA proteins in Clade 2, indicating the selected amino acids may be important for the functional divergence of proteins within Clade 2 [41]. The extensive colinear relationship among GASA genes of dicotyledonous species has also been observed in previous studies [3]. However, the lack of colinear GASA gene pairs between *A. thaliana* and *O. sativa* may suggest rounds of chromosomal rearrangements during the independent evolution of *O. sativa* after monocotyledonous plant speciation.

The promoter analysis revealed many hormonal responsive cis-acting elements within *PmGASA* promoters. We detected abundant ABA responsive ABRE-element, auxin responsive elements (AuxRR-core/TGA), GA responsive elements (GARE/TATC-box/P-box), SA responsive elements (TCA), and MeJA responsive elements (TGACG/CGTCA) present within the promoter region of different *PmGASA* members. Among them, all three types of elements (GA, ABA, and IAA responsive elements) were present in only *PmGASA1* and *PmGASA11* at the same time. The expression analysis confirmed the results that *PmGASA1* and *PmGASA11* were responsive to exogenous treatments of IAA, GA, and ABA. The RT-PCR assays revealed GA inducibility in most *PmGASA* genes. Interestingly, *PmGASA6*, *PmGASA7*, and *PmGASA14*, lacking GA responsive element within their promoters, also displayed induced or repressed expression patterns within three hours after GA treatment. It is possible that these *PmGASA* genes are regulated indirectly by GA responsive regulators. In addition to *PmGASA1* and *PmGASA11*, the expression patterns of *PmGASA2*, *PmGASA5*, *PmGASA6*, *PmGASA7*, *PmGASA13*, *PmGASA14*, and *PmGASA15* all displayed responsiveness to exogenous GA, ABA, and IAA treatments, indicating their possible role in integrating the gibberellin acid-, abscisic acid- and auxin-signaling pathways in *P. mume*.

The transcription regulatory network analysis of *PmGASA* family genes also predicted a number of TFs related to hormonal signaling. For example, RGA1 encodes a GARS family transcription factor that may act upstream of *PmGASA1*, *PmGASA13*, *PmGASA14*, *PmGASA15*, and *PmGASA16* in repressing gibberellin responses. ABF2 [42], ABI5 [43], and AREB3 [43] are three transcription factors known to regulate the ABA-mediated seed germination, development, and maturation in *Arabidopsis* [42,43]. These three TFs may regulate *PmGASA7* and *PmGASA12* transcription through binding to ABRE-motifs during ABA signaling pathways in *P. mume*. ARFs is a family of transcription factors that specifically binds to the 5′-TGTCTC-3′ element of auxin-responsive genes to regulate early auxin responses [44]. *ARF10* and *ARF16* were predicted to regulate the transcription of *PmGASA7* through binding to its AuxRR-core motifs in *P. mume*. These results suggested that *PmGASA* genes are possibly involved in a wide range of plant hormone signal transduction networks. Additionally, the abiotic stress responsive cis-elements were detected within *PmGASA* promoters, indicating the possible involvement of *PmGASA* genes in relevant biological processes.

In addition to hormonal responses, GASA genes are also known to play important roles in plant organ growth and development. To further elucidate the functional role of *PmGASA* genes, we examined the expression patterns of *GASA* genes across different plant organs. The *PmGASA* genes displayed distinct spatial expression patterns across stem, leaf, root, fruit, and flower bud tissues. *PmGASA5*, *PmGASA8*, and *PmGASA10* were specifically expressed in flowers; *PmGASA1*, *PmGASA3*, *PmGASA11*, and *PmGASA14* were relatively high-expressed in fruits; and *PmGASA2*, *PmGASA6*, *PmGASA7*, *PmGASA9*, *PmGASA12*, *PmGASA13*, and *PmGASA15* were mostly transcribed in stems. We failed to detect the transcript of *PmGASA4* and *PmGASA16* in any of the five plant organs. The divergent tissue-specific expression patterns of *PmGASAs* may suggest their functional divergence in regulating the development and formation of different organs in *P. mume*.

Bud dormancy cycling is an adaptive process crucial to the survival of temperate tree species during harsh winters [45]. Perennial tree species usually enter endodormancy upon exposure to short day or low temperatures after growth cessation and bud set [46]. When buds accumulate sufficient chilling, endodormancy is released and trees enter ecodormancy [47]. Gibberellic acid is a class of plant hormones involved in regulating bud dormancy establishment and dormancy release in perennial trees [48]. During endodormancy release, the long-term chilling induces GA biosynthesis, which mediates the transcription of *FT* and promotes bud flush [49]. Induced by gibberellins, GASA family genes may play important roles in regulating hormonal signal transduction during bud dormancy cycling. In white pear (*Pyrus pyrifolia*), *PpyGAST1* is highly induced during endodormancy release [19]. The over-expression of *PpyGAST1* in *Arabidopsis* leads to early seed germination through up-regulating GA biosynthetic genes *AtGA20OX2* and *AtGA3OX1*, and the downstream *AtEXPA1*, indicating the functional role of *PpyGAST1* in promoting dormancy release [19]. However, whether GASA gene members are involved in regulating floral bud flush and dormancy release in other perennial fruit trees is still unknown. Therefore, we analyzed the *PmGASA* expression patterns during floral bud development. We observed strong induction of *PmGASA16* after endodormancy exit in floral buds, indicating its possible role in promoting the endodormancy break in *P. mume*. We also observed significant up-regulation of *PmGASA1*, *PmGASA5*, *PmGASA7*, *PmGASA9*, *PmGASA10*, *PmGASA11*, *PmGASA13*, *PmGASA14*, and *PmGASA15* during flower bud flush. These *PmGASA* genes are likely regulated by hormonal level change or may be responsible for modulating related hormonal responses during floral organ development and bud flushing in *P. mume* [50]. The repression of *PmGASA2*, *PmGASA6*, and *PmGASA12* during the whole process of floral bud break may be required for the complex developmental process occurring in the flushing flower bud.

## 4. Materials and Methods

### 4.1. Identification of GASA Genes in Six Plant Species

We first obtained the genomes of *Arabidopsis thaliana*, *Malus domestica*, *Prunus mume*, *Prunus persica*, *Populus tricocarpa*, and *Oryza sativa* from public datasets including TAIR (http://www.arabidopsis.org), GDR (https://www.rosaceae.org), and Phytozome (https://phytozome-next.jgi.doe.go) (accessed all databases on 24 June 2022). To identify GASA family genes in each species, we used the Hidden Markov Model of the GASA domain (PF02704) as a query to search for all putative GASA proteins using the HMMER v3.3.2 software (https://www.ebi.ac.uk/Tools/hmmer/; accessed on 4 September 2022) [51]. On the other hand, the protein sequences of 15 AtGASA genes were used to blast against the genomes of five other species (*M. domestica*, *P. mume*, *P. persica*, *Populus tricocarpa*, and *O. sativa*), and the top hits with e-value ≤ 1.0 × 10^−10^ were considered as candidate genes. By combining all GASA candidate genes identified using HMMER and BLASTP approaches, we verified the presence and completeness of the GASA domain in each GASA protein using NCBI CDD search (https://www.ncbi.nlm.nih.gov/Structure/cdd/wrpsb.cgi; accessed on 24 June 2022) and the Simple Modular Architecture Research Tool (SMART: http://smart.embl.de/; accessed on 24 June 2022) [40]. Only the non-redundant genes with complete GASA domains were included in the following analysis.

### 4.2. Physicochemical Property Analysis of PmGASA Proteins

The physicochemical characteristics of PmGASA proteins were analyzed using the ExPASy online tool (http://web.expasy.org/protparam/; accessed on 3 July 2022), including the isoelectric point (pI), number of amino acids, and molecular weight (MW) [52]. The protein tertiary structures and transmembrane helices were predicted using the PHYRE2 program (https://www.genscript.com/wolf-psort.html; accessed on 3 July 2022) and TMHMM server v2.0 (http://www.cbs.dtu.dk/services/TMHMM-2.0/; accessed on 3 July 2022). Finally, the protein subcellular locations were predicted using the Plant-mPLoc program (http://www.csbio.sjtu.edu.cn/bioinf/plant-multi/; accessed on 3 July 2022) [53].

### 4.3. Gene Structure and Protein Motif Analysis of PmGASA Genes

The genome annotation information was retrieved for *PmGASA* family genes. The exon/intron arrangement and chromosome locations of *PmGASA* genes were visualized using TBtools software [54]. The conserved protein motifs of PmGASAs were analyzed using MEME (https://meme-suite.org/meme/tools/meme; accessed on 4 July 2022) [55] with their sequences generated and visualized using WebLogo platform (http://weblogo.berkeley.edu/logo.cgi; accessed on 4 July 2022) [56]. All genes were renamed according to their positions across chromosomes.

### 4.4. Phylogenetic Analysis of GASA Family Genes

Multiple sequence alignment was performed by aligning the whole protein sequences of the GASA family genes with ClustalW within the MEGA 11.0 software (www.megasoftware.net) [57]. Subsequently, a phylogenetic tree of GASA family genes among *A. thaliana*, *M. domestica*, *P. mume*, *P. persica*, *Populus tricocarpa*, and *O. sativa* was constructed with MEGA 11.0 using the neighbor-joining (NJ) method with 1000 bootstrap replicates. Similarly, the phylogenetic tree of *PmGASAs* was built following the same protocol. The phylogenetic tree was finally visualized using the iTOL online tool (http://itol2.embl.de; accessed on 5 July 2022). We also extracted the amino acids within the GASA domains of PmGASA proteins and aligned them with ClustalW. The amino acid sequence alignment was finally visualized using GeneDoc software [58].

### 4.5. Microsynteny and Selection Analysis of GASA Family Genes

To identify the syntenic relationship among GASA family genes, we first performed all-against-all BLASTP among genomes of six investigated plant species. Based on the top BLASTP hits (e-value < 1.0 × 10^−10^), we detected the GASA gene pairs located within the colinear genome blocks across species using MCScanX [59]. The duplicated GASA family genes were detected based on the self-blast approach, followed by duplication event detection using ‘duplicate_gene_classifier’ implemented in MCscanX [59]. The intra-species and inter-species synteny among GASA family genes were visualized using the software TBtools [54]. Non-synonymous (Ka) and synonymous (Ks) substitution ratios were calculated for duplicated *PmGASA* gene pairs by aligning their protein sequences using ClustalW followed by the PAL2NAL program (http://www.bork.embl.de/pal2nal/; accessed on 6 August 2022). The mode of selection (positive, purifying, or neutral) acting on the duplicated gene pairs was determined with Ka/Ks ratio >1, <1, or =1, respectively. We also evaluated the selection pressure among codons of *PmGASA* genes with the software Selecton (http://selecton.tau.ac.il; accessed on 6 August 2022) [60].

### 4.6. Cis-Acting Element Analysis of PmGASA Genes

The promoter sequences of *PmGASA* genes were extracted as the 2kb sequence upstream of the start codon for each gene based on the genome sequences of *P. mume*. The cis-elements were predicted and analyzed using PlantCARE (http://bioinformatics.psb.ugent.be/webtools/plantcare/html/; accessed on 24 July 2022). The cis-regulatory elements related to hormonal responses and abiotic stress were further classified.

### 4.7. Tissue Specific Expression of PmGASA Genes

To understand the expression pattern of *PmGASA* genes across different plant organs, we obtained the tissue transcriptome data of *P. mume* (GSE4760162) from the NCBI SRA database [26]. Raw sequencing reads were preprocessed with Trimmomatic software, followed by aligning clean-paired reads to their corresponding reference genomes using HISAT2 [40]. The FPKM values were obtained for GASA genes and were compared across different tissues using the R package ‘edgeR’ [61]. The expression patterns were visualized using the ‘pheatmap’ package in R. Hierarchical clustering was performed on expression profiles of *PmGASAs* based on the Euclidean distance matrix using ‘hclust’ in R (with agglomeration method set as ‘ward.D’).

### 4.8. Expression Pattern of PmGASA Genes during Floral Bud Development

To investigate the functional roles of *PmGASAs* during floral bud break, we retrieved the RNA-seq data of flower buds at four developmental stages, including endodormancy, ecodormancy, bud break, and bud flush [62]. The raw data were cleaned, analyzed, and normalized to FPKM values following the pipeline described above. The expression profiles of *PmGASAs* during the floral bud development process were obtained and visualized with heatmap using R.

### 4.9. Plant Materials and qRT-PCR Analysis of PmGASA Genes in Gibberellin Treatment

A five-year-old *P. mume* tree cultivated at Beijing Forestry University (China) was used in this study. We performed hormonal treatments by applying 100 mM GA_3_, 100 mM IAA, or 300 mM ABA to newly sprouted leaves and dormant floral buds. The flower buds of ecodormant stage and young leaves from current-year branches were mixed and collected at 0, 1, 3, 6, 12, 24, and 48 h after hormonal treatments. Samples were immediately frozen with liquid nitrogen and stored at −80 °C.

Total RNA was extracted from plant tissue samples with a Plant RNA Kit (Omega Bio-tek, Norcross, GA, USA) following the manufacturer’s protocols, and the integrity was verified using 1% agarose gel electrophoresis. The cDNA was synthesized using the PrimeScript RT reagent kit (Takara, Kusatsu, Japan). The relative expression of GASA genes was assessed with qRT-PCR assays, with three technical replicates with standard deviations assessed. The qRT-PCR experiments were performed on the PikoReal real-time PCR platform (Thermo Fisher Scientific, Waltham, MA, USA) with temperature settings: 95 °C for 30 s, 40 cycles of 95 °C for 5 s, 60 °C for 30 s, and 72 °C for 30 s. The protein phosphatase 2A (PP2A) gene was used as an internal reference to calculate the relative gene expression levels using the 2^−ΔΔCt^ method [29]. The primers used for qRT-PCR experiments are provided in Table S1.

### 4.10. Transcriptional Regulatory Network Prediction of PmGASAs

Putative transcription factors (TFs) regulating *PmGASA* genes were predicted using the Plant Transcriptional Regulatory Map (PTRM) website (http://plantregmap.gao-lab.org/regulation_prediction.php; accessed on 18 August 2020) at the threshold *p*-value  ≤  1 × 10^−6^ with *PmGASA* gene promoter sequences as input [63]. The TF-gene regulatory network was visualized using Cytoscape software [64].

## 5. Conclusions

In our study, we identified a total of 16 *PmGASA* genes in the *P. mume* genome and analyzed their chromosomal location, gene structure, protein physicochemical characteristics, and conserved motifs. The phylogenetic analysis revealed three major gene clades among *PmGASAs*. With promoter analysis, we identified various cis-acting regulatory elements and constructed the putative transcriptional regulatory networks involving *PmGASAs*. By combing synteny analysis and selection analysis, we inferred the expansion and evolutionary trajectory of the GASA family genes in *P. mume*. Furthermore, we analyzed the spatial and temporal expression patterns of *PmGASA* genes across different plant organs, during floral bud break and hormonal responses. Overall, these findings suggested the possible involvement of *PmGASAs* in mediating floral bud break and hormonal signaling in *P. mume*. Our study provided insights into the evolution and functional implication of GASA family genes in *P. mume* and has laid theoretical basis for future studies exploring the molecular mechanism of *GASA* genes in other woody perennial trees.

## Figures and Tables

**Table 1:** Detailed information of GASA family genes identified in *P. mume*. For each gene, their Gene_ID (gene identifier in the published genome of *P. mume*), NCBI accession (closest gene accession name returned from NCBI BLASTP), the start and end coordinate of GASA domain, HMM accession ID, domain name, and e-value for each PmGASA protein query in the Pfam database are listed.

Gene_ID	Gene_Name	NCBI Accesion	Start	End	HMM Accession	HMM Name	E-Value (Pfam)
Pm005363	*PmGASA1*	XP_007223838.1	29	88	PF02704	GASA	4.29 × 10^−26^
Pm006215	*PmGASA2*	CAB4265575.1	50	109	PF02704	GASA	3.41 × 10^−27^
Pm009495	*PmGASA3*	XP_008224510.1	35	92	PF02704	GASA	1.44 × 10^−26^
Pm009496	*PmGASA4*	XP_008224511.1	36	95	PF02704	GASA	8.24 × 10^−25^
Pm014337	*PmGASA5*	XP_007215190.1	35	94	PF02704	GASA	1.19 × 10^−24^
Pm015762	*PmGASA6*	ONI19434.1	159	219	PF02704	GASA	1.76 × 10^−26^
Pm015883	*PmGASA7*	PQQ10068.1	49	108	PF02704	GASA	2.24 × 10^−18^
Pm021248	*PmGASA8*	KAH0977881.1	9	62	PF02704	GASA	1.88 × 10^−13^
Pm021249	*PmGASA9*	CAB4290623.1	13	60	PF02704	GASA	2.52 × 10^−26^
Pm022352	*PmGASA10*	XP_008237395.1	54	113	PF02704	GASA	1.05 × 10^−26^
Pm024681	*PmGASA11*	XP_008239716.1	62	122	PF02704	GASA	3.03 × 10^−27^
Pm024909	*PmGASA12*	XP_008239947.1	48	107	PF02704	GASA	6.36 × 10^−26^
Pm025746	*PmGASA13*	XP_008240742.1	57	116	PF02704	GASA	5.82 × 10^−31^
Pm026754	*PmGASA14*	XP_016651693.1	49	108	PF02704	GASA	8.88 × 10^−24^
Pm029238	*PmGASA15*	XP_008244953.1	53	112	PF02704	GASA	2.15 × 10^−30^
Pm030034	*PmGASA16*	XP_008245155.1	46	106	PF02704	GASA	2.75 × 10^−23^

**Table 2:** Protein physiochemical analysis of GASA family genes identified in *P. mume*.

Gene_Name	Protein Length (aa)	MW (Da)	pI	Instability Index	Aliphatic Index	GRAVY	Subcellular Prediction
*PmGASA1*	88	9613.29	8.58	35.77	59.77	−0.064	Golgi; Nucleus
*PmGASA2*	109	11,962.01	8.28	83.81	65.23	−0.353	Nucleus
*PmGASA3*	120	12,321.77	8.9	50.46	28.67	−0.911	Nucleus
*PmGASA4*	95	10,646.52	8.61	56.36	56.53	−0.28	Golgi
*PmGASA5*	94	10,595.56	9.09	43.35	61.17	−0.181	Golgi
*PmGASA6*	219	23,383.3	10	72.24	87.72	−0.058	Nucleus
*PmGASA7*	108	11,732.75	9.26	42.49	47.13	−0.276	Golgi
*PmGASA8*	108	12,081.8	8.12	76.86	44.26	−0.867	Golgi; Nucleus
*PmGASA9*	183	20,356.95	6.06	46.45	62.4	−0.426	Nucleus
*PmGASA10*	113	12,489.76	9.58	57.15	62.3	−0.358	Golgi
*PmGASA11*	122	13,454.97	9.16	43.61	79.02	−0.018	Nucleus
*PmGASA12*	107	11,856.05	9.11	39.51	57.48	−0.176	Golgi
*PmGASA13*	116	12,872.11	9.11	40.8	55.52	−0.342	Nucleus
*PmGASA14*	108	11,611.52	8.92	61.23	53.43	−0.188	Golgi
*PmGASA15*	112	12,392.59	9.02	39.73	58.39	−0.178	Golgi
*PmGASA16*	106	11,785.77	8.89	44.91	71.7	−0.163	Golgi; Nucleus

**Table 3:** The Ka/Ks ratios of paralogous gene pairs of *PmGASA* gene family.

Gene 1	Gene 2	Ka	Ks	Ka/Ks	Selection Pressure	Gene Duplications
*PmGASA3*	*PmGASA4*	0.36	1.17	0.31	Purifying	Tandem
*PmGASA1*	*PmGASA8*	0.39	8.17	0.05	Purifying	WGD or Segmental
*PmGASA2*	*PmGASA14*	0.41	1.67	0.24	Purifying	WGD or Segmental
*PmGASA6*	*PmGASA11*	0.43	3.39	0.13	Purifying	WGD or Segmental
*PmGASA7*	*PmGASA12*	0.16	0.92	0.18	Purifying	WGD or Segmental

## Data Availability

The datasets used in this study are publicly available. All analyzed data can be found in the article or in the supplementary material.

## Supplementary Materials

The supporting information can be downloaded at: https://www.mdpi.com/article/10.3390/ijms231810923/s1.
