# Supplementary material for: Genome-Wide Identification, Evolution, and Expression Analysis of GASA Gene Family in Prunus mume
Source: Int J Mol Sci. 2022 Sep 18;23(18):10923. doi: 10.3390/ijms231810923 (PMC9506367; doi:10.3390/ijms231810923)
Supplement: Supplementary file 1 [file ijms-23-10923-s001.zip › ijms-1907475-supplementary.pdf]

Figure S1. Chromosomal location of *PmGASA* genes. Chromosome and scaffold sizes are indicated by their relative length.

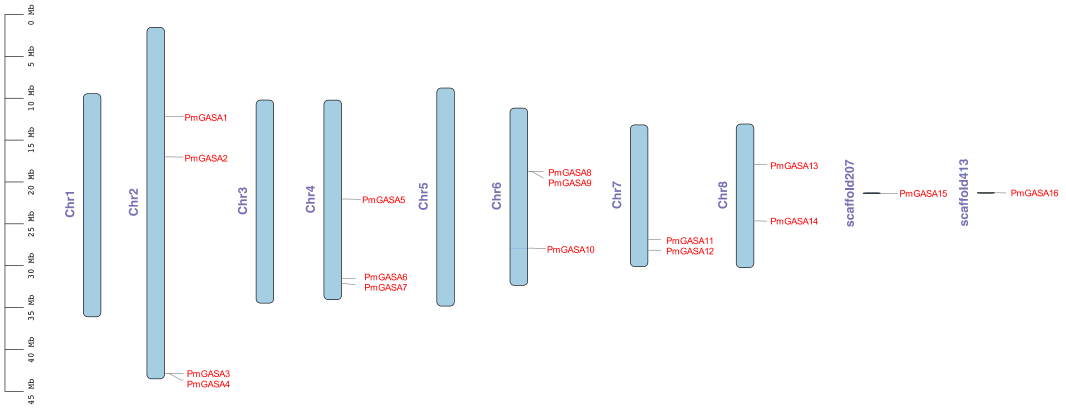

Figure S2. Featured motifs of PmGASA proteins.

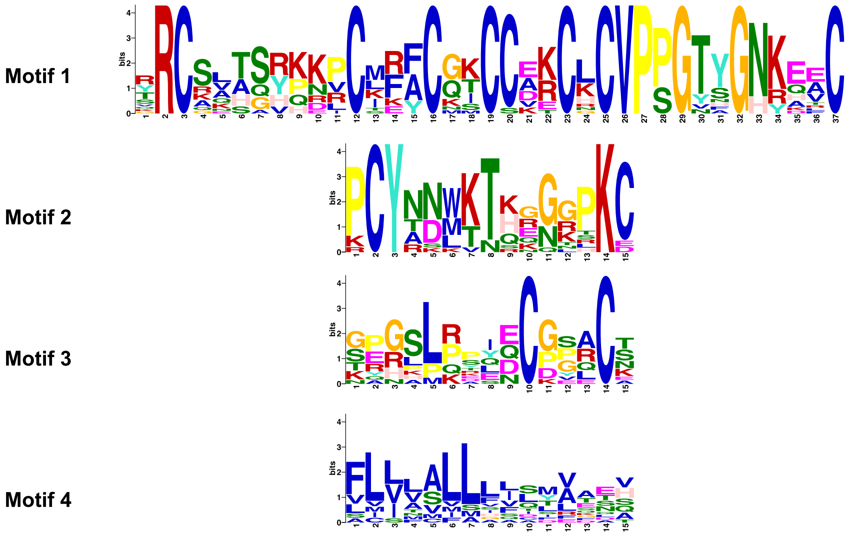

Figure S3. Synteny analysis of *GASA* genes identified in *P. mume*. The rings represent different chromosomes and scaffolds. The grey blocks represent syntenic regions and the red lines represent collinear *GASA* gene pairs.

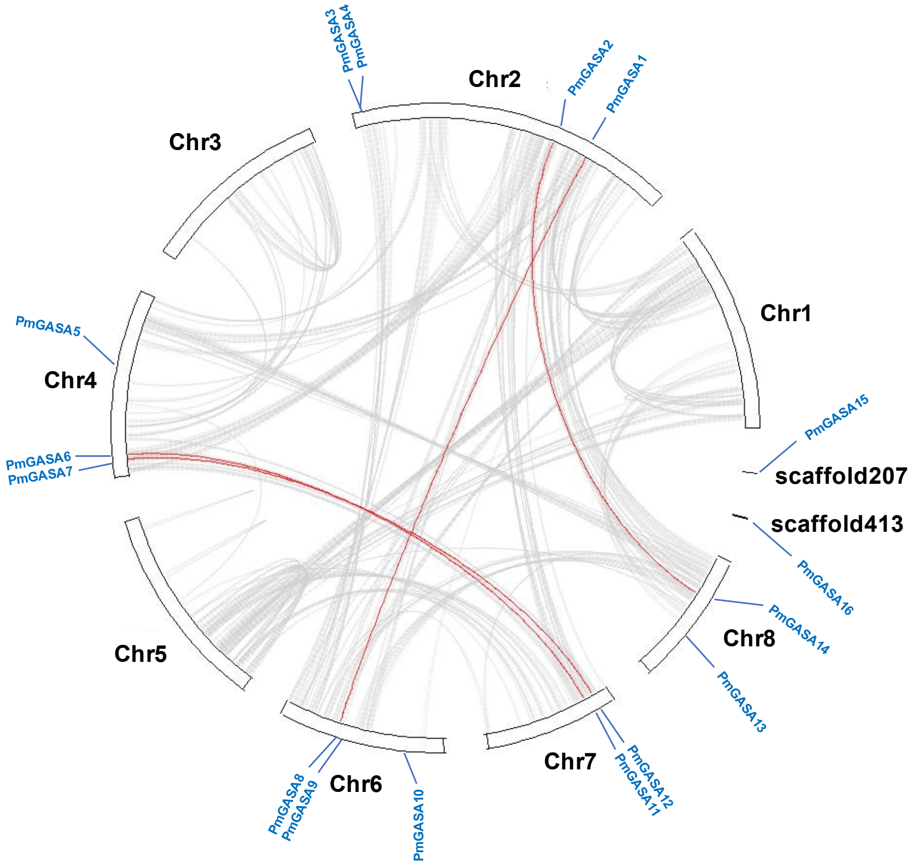

Figure S4. Selective pressure analysis of three *PmGASA* gene clades identified positively selected sites. Amino acids in yellow shades are under positive selection and those in purple shades are under purifying selection. Amino acids were colored accordingly for clade 1, clade 2, and clade 3 with *PmGASA3*, *PmGASA9*, *PmGASA6* as reference genes, respectively.

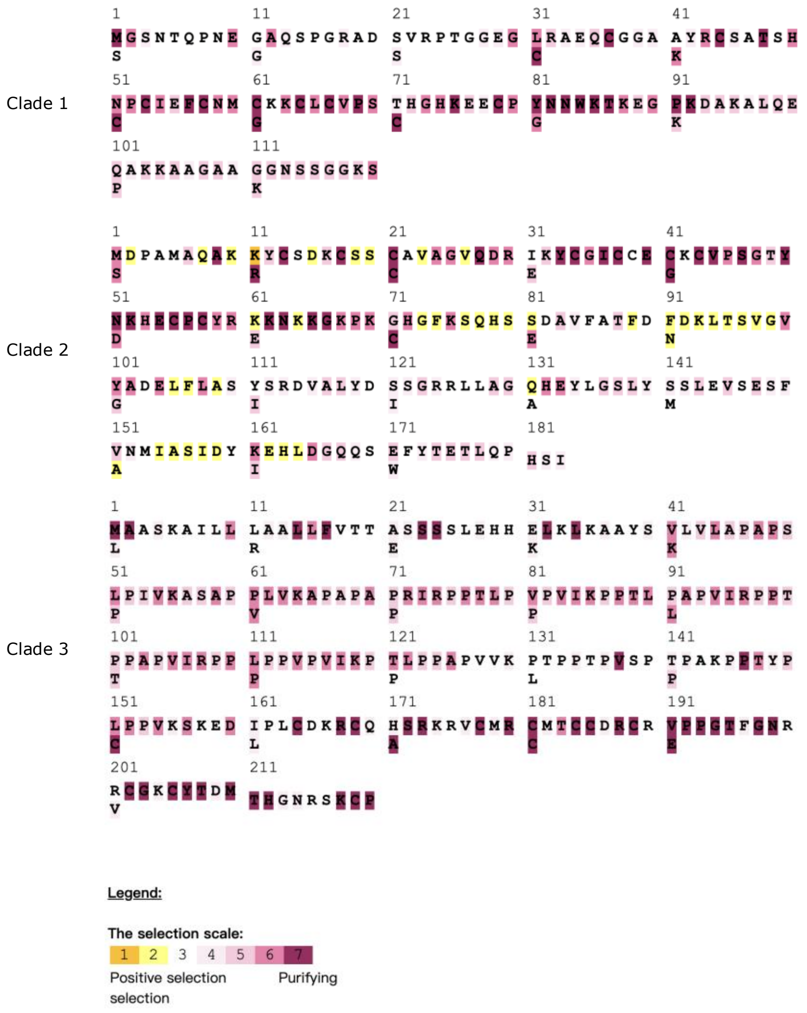

Figure S5. The putative transcriptional regulatory network of the *PmGASA* genes constructed based on Plant Transcriptional Regulatory Map (PTRM) database. Arrows represent the regulatory relationships between *PmGASAs* and their predicted upstream transcription factors.

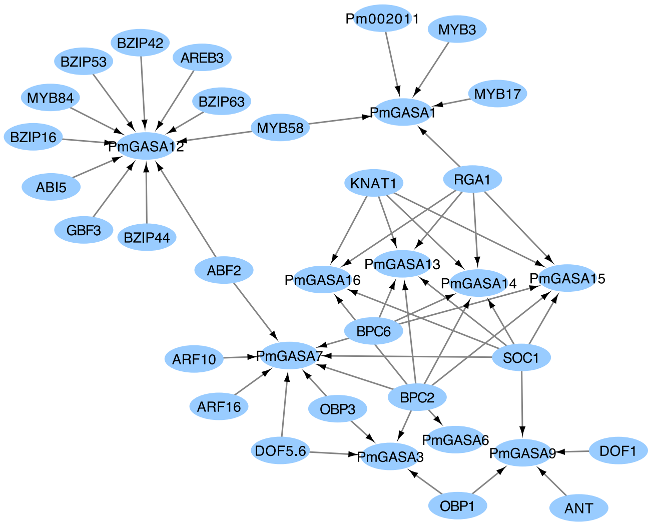

Figure S6. Relative expression levels of *PmGASAs* in floral buds after GA treatment. Error bars represent the standard deviations assessed from three technical replicates.

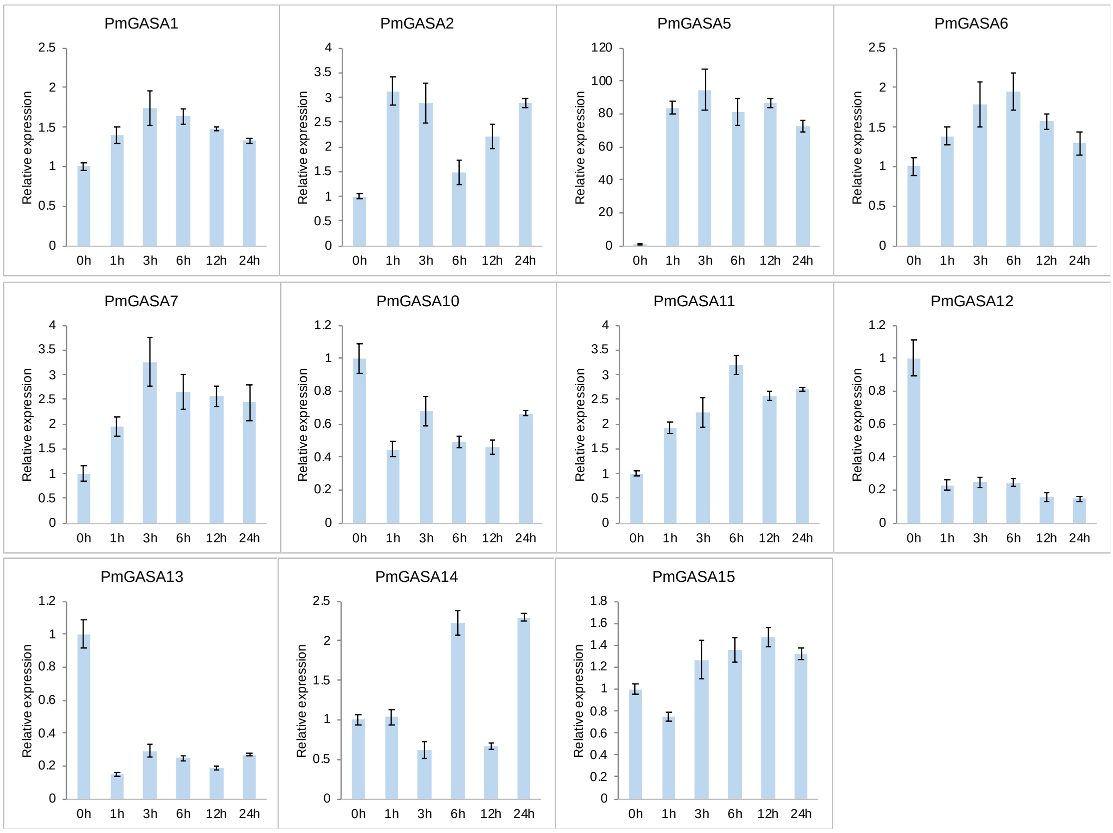

Table S1. The primers of *PmGASA* genes used in the qRT-PCR assays.

Gene	Primer	Sequence
PmGASA1	Pm005363-F	GAGCGGTGCTTGAAGTACTGTG
	Pm005363-R	TTGGAGTTTTTGAGGTCCCTGT
PmGASA2	Pm006215-F	ATGGCCAAGAAGATGATGTTGA
	Pm006215-R	GGTCACCGTTTCATGAGAATCA
PmGASA3	Pm009495-F	TGCTCAGCAACTTCACACAAGA
	Pm009495-R	TGTGCCTGATGGAACACATAGA
PmGASA4	Pm009496-F	CACTTCGTCGTGAAGATTGTCC
	Pm009496-R	ACAAAGACATTTGTCGCAGCAT
PmGASA5	Pm014337-F	TCTCTGATGTGGCTGAGGGTTA
	Pm014337-R	CATTTTTGGCAGAAGAACATGC
PmGASA6	Pm015762-F	CACTCCACCAACTCCTGTCAGT
	Pm015762-R	CTCTTGTCACACAAAGGGATGC
PmGASA7	Pm015883-F	TACCACAAGCCTTGCATGTTCT
	Pm015883-R	TGGGTCTTCCAGTTGTTGTAGC
PmGASA8	Pm021248-F	TGTGTGCCTTCTGGGAATTATG
	Pm021248-R	TCGTTGAGCACTTGTCATTGTG
PmGASA9	Pm021249-F	GCTTGCAGGAATTCAACATGAG
	Pm021249-R	CATCAAGGTGTTCCTTGGCATA
PmGASA10	Pm022352-F	CAGTGATGCTGCGAATTCTACC
	Pm022352-R	AGCATGCATAGTCGCAATTGAT
PmGASA11	Pm024681-F	TGGTTCTTTTGCTGGGAGTTTT
	Pm024681-R	TGCGCAAGATGAGGTAATTGTT
PmGASA12	Pm024909-F	ATGGTTGCAACACAGGTTATGG
	Pm024909-R	GCTCTTTAGACTCCCAGGACCA
PmGASA13	Pm025746-F	GCTCTCCCTCGTTATGCTTCAT
	Pm025746-R	AATTCCATTCATCGTGTGGTTG
PmGASA14	Pm026754-F	GATGATGGCTTCCCTTGTTTTC
	Pm026754-R	GCTCCATTGTTGTTCATCGTGT
PmGASA15	Pm029238-F	CCTTGTCTGTTTTTCTGCCAAA
	Pm029238-R	TGGTCCTCCTCTCTTGGTCTTC
PmGASA16	Pm030034-F	GTTATGGCGGTTTTGCTCTTCT
	Pm030034-R	CTTCTGTTTGCTCCTTTCACCA

Table S2. Detailed information of GASA family genes identified in *A. thaliana*, *M. domestica*, *Populus tricocarpa*, *O. sativa*, and *Prunus persica*.

Gene_ID	Gene name	NCBI accession	Start	End	HMM accession	HMM name	E-Value (Pfam)
AT1G75750.1	AtGASA1	NP_565116.1	39	98	PF02704	GASA	1.79E-22
AT5G59845.1	AtGASA10	NP_568914.1	30	89	PF02704	GASA	4.51E-27
AT2G18420.1	AtGASA11	NP_179433.2	35	94	PF02704	GASA	2.32E-23
AT2G30810.1	AtGASA12	NP_180639.2	47	106	PF02704	GASA	4.78E-24
AT3G10185.1	AtGASA13	A8MR46.1	44	103	PF02704	GASA	2.10E-21
AT5G14920.1	AtGASA14	KAG7609230.1	215	275	PF02704	GASA	6.44E-26
AT1G10588.1	AtGASA15	NP_001077503.1	31	90	PF02704	GASA	7.11E-25
AT4G09610.1	AtGASA2	NP_192699.1	40	99	PF02704	GASA	2.49E-19
AT4G09600.1	AtGASA3	NP_192698.1	40	99	PF02704	GASA	6.40E-26
AT5G15230.1	AtGASA4	NP_197027.1	47	106	PF02704	GASA	5.21E-27
AT3G02885.1	AtGASA5	NP_566186.1	38	97	PF02704	GASA	2.12E-19
AT1G74670.1	AtGASA6	NP_177605.2	42	101	PF02704	GASA	7.49E-29
AT2G14900.1	AtGASA7	CAD5318533.1	50	108	PF02704	GASA	1.21E-23
AT2G39540.1	AtGASA8	NP_181486.1	29	87	PF02704	GASA	2.44E-27
AT1G22690.1	AtGASA9	NP_001185065.1	60	119	PF02704	GASA	3.76E-26
MD00G1092500	MdGASA1	XP_028953983.1	45	104	PF02704	GASA	3.99E-25
MD09G1038100	MdGASA10	XP_028963601.1	202	262	PF02704	GASA	5.07E-17
MD09G1038300	MdGASA11	XP_008379287.2	94	154	PF02704	GASA	1.53E-13
MD09G1051300	MdGASA12	XP_018507357.2	49	108	PF02704	GASA	8.02E-20
MD09G1263900	MdGASA13	XP_008381340.1	36	94	PF02704	GASA	1.74E-17
MD13G1086500	MdGASA14	XP_008389581.1	48	108	PF02704	GASA	5.55E-22
MD13G1285300	MdGASA15	XP_008367840.1	51	110	PF02704	GASA	1.96E-25
MD14G1081900	MdGASA16	XP_008392374.1	57	116	PF02704	GASA	7.24E-31
MD14G1200300	MdGASA17	XP_008393156.1	48	107	PF02704	GASA	4.85E-27
MD15G1012300	MdGASA18	XP_008387949.1	48	107	PF02704	GASA	4.35E-24
MD15G1012400	MdGASA19	XP_017191904.1	6	65	PF02704	GASA	3.63E-22
MD02G1132300	MdGASA2	XP_028946449.1	47	106	PF02704	GASA	1.88E-23
MD15G1012500	MdGASA20	XP_028951122.1	48	107	PF02704	GASA	1.39E-23
MD15G1012600	MdGASA21	XP_017191904.1	6	65	PF02704	GASA	6.08E-22
MD15G1073900	MdGASA22	XP_008360977.1	29	88	PF02704	GASA	4.24E-27
MD15G1246100	MdGASA23	RXH75020.1	64	123	PF02704	GASA	1.18E-26
MD16G1086000	MdGASA24	XP_008353009.1	47	107	PF02704	GASA	1.31E-23
MD17G1039200	MdGASA25	XP_008353408.1	115	175	PF02704	GASA	1.24E-24
MD17G1039400	MdGASA26	XP_008353409.2	105	165	PF02704	GASA	1.48E-20
MD17G1041500	MdGASA27	XP_009341013.1	49	108	PF02704	GASA	2.05E-19
MD17G1259200	MdGASA28	XP_008353837.1	36	94	PF02704	GASA	3.32E-17
MD02G1132400	MdGASA3	TQD80925.1	47	106	PF02704	GASA	4.10E-24
MD04G1009200	MdGASA4	XP_028957554.1	55	114	PF02704	GASA	2.16E-24

MD04G1009300	MdGASA5	XP_008380555.1	55	114	PF02704	GASA	2.69E-24
MD05G1093700	MdGASA6	RXH97527.1	30	88	PF02704	GASA	8.46E-25
MD08G1088900	MdGASA7	XP_028962285.1	29	88	PF02704	GASA	4.63E-27
MD08G1089100	MdGASA8	XP_028962286.1	29	88	PF02704	GASA	1.73E-27
MD08G1089300	MdGASA9	XP_008377985.1	29	88	PF02704	GASA	4.48E-27
LOC_Os03g14550.1	OsGASA1	ABF94978.1	42	101	PF02704	GASA	3.75E-28
LOC_Os09g24840.2	OsGASA10	XP_015611416.1	53	112	PF02704	GASA	3.67E-29
LOC_Os10g02625.1	OsGASA11	XP_015613996.1	45	104	PF02704	GASA	6.98E-30
LOC_Os03g55290.1	OsGASA2	XP_015629016.1	31	93	PF02704	GASA	1.93E-22
LOC_Os04g39110.1	OsGASA3	XP_015635047.1	45	105	PF02704	GASA	6.85E-19
LOC_Os04g39110.2	OsGASA4	KAF2934394.1	14	74	PF02704	GASA	1.58E-19
LOC_Os05g31280.1	OsGASA5	XP_015638982.1	93	152	PF02704	GASA	1.13E-27
LOC_Os05g35690.1	OsGASA6	XP_015638298.1	33	92	PF02704	GASA	4.85E-28
LOC_Os06g15620.1	OsGASA7	XP_015641724.1	51	110	PF02704	GASA	3.59E-25
LOC_Os07g40240.1	OsGASA8	XP_015646546.1	43	102	PF02704	GASA	6.38E-14
LOC_Os09g24840.1	OsGASA9	XP_015611416.1	53	112	PF02704	GASA	3.67E-29
Prupe.1G008600.1	PpGASA1	ONI26189.1	49	108	PF02704	GASA	6.08E-25
Prupe.4G257500.1	PpGASA10	XP_020416901.1	53	112	PF02704	GASA	3.19E-30
Prupe.5G178400.1	PpGASA11	XP_007211041.1	50	110	PF02704	GASA	1.50E-26
Prupe.5G193400.1	PpGASA12	XP_007209788.1	48	107	PF02704	GASA	3.45E-27
Prupe.7G049000.1	PpGASA13	XP_007216796.2	57	116	PF02704	GASA	2.64E-30
Prupe.7G168200.1	PpGASA14	ONH97069.1	97	156	PF02704	GASA	6.02E-26
Prupe.8G058200.1	PpGASA15	XP_007221450.2	54	113	PF02704	GASA	1.60E-26
Prupe.8G138400.1	PpGASA16	ONH91835.1	34	83	PF02704	GASA	3.94E-14
Prupe.8G138600.1	PpGASA17	XP_007200798.1	33	80	PF02704	GASA	2.82E-17
Prupe.1G260200.1	PpGASA2	ONI30584.1	46	106	PF02704	GASA	2.69E-23
Prupe.1G365600.1	PpGASA3	XP_007225927.1	48	107	PF02704	GASA	5.89E-26
Prupe.1G365700.1	PpGASA4	XP_034200285.1	50	109	PF02704	GASA	4.87E-26
Prupe.1G426700.1	PpGASA5	XP_007223838.1	29	88	PF02704	GASA	5.00E-26
Prupe.3G161500.1	PpGASA6	XP_007215190.1	35	94	PF02704	GASA	4.16E-26
Prupe.3G267600.1	PpGASA7	PQQ10068.1	49	108	PF02704	GASA	3.78E-19
Prupe.3G279500.1	PpGASA8	ONI19427.1	86	145	PF02704	GASA	3.18E-12
Prupe.3G279800.1	PpGASA9	ONI19434.1	150	210	PF02704	GASA	6.44E-22
Potri.001G254100.2.p	PtGASA1	XP_002298301.3	54	113	PF02704	GASA	2.75E-27
Potri.006G044400.1.p	PtGASA10	XP_011027306.1	57	116	PF02704	GASA	3.06E-26
Potri.007G051300.1.p	PtGASA11	KAH8503286.1	43	102	PF02704	GASA	1.97E-14
Potri.009G092600.1.p	PtGASA12	XP_002313889.2	30	89	PF02704	GASA	1.32E-22
Potri.012G076700.1.p	PtGASA13	XP_002318020.1	49	109	PF02704	GASA	7.10E-27
Potri.013G113400.3.p	PtGASA14	RQO99302.1	32	91	PF02704	GASA	1.10E-28
Potri.014G020100.1.p	PtGASA15	XP_006374850.1	29	88	PF02704	GASA	3.52E-28
Potri.015G071500.1.p	PtGASA16	XP_002321566.1	49	109	PF02704	GASA	4.42E-26
Potri.017G083000.1.p	PtGASA17	AII82562.1	48	107	PF02704	GASA	5.12E-22
Potri.017G124200.1.p	PtGASA18	XP_011021371.1	36	95	PF02704	GASA	9.95E-27

Potri.019G083900.1.p	PtGASA19	XP_002325557.2	51	110	PF02704	GASA	3.85E-24
Potri.001G297700.1.p	PtGASA2	KAG6793118.1	29	88	PF02704	GASA	1.63E-22
Potri.001G315500.1.p	PtGASA3	PNT57691.1	37	96	PF02704	GASA	2.23E-28
Potri.001G350600.3.p	PtGASA4	XP_011047278.1	185	245	PF02704	GASA	4.65E-23
Potri.002G022500.1.p	PtGASA5	XP_002300706.2	59	118	PF02704	GASA	2.20E-29
Potri.002G022600.2.p	PtGASA6	XP_002300707.1	43	102	PF02704	GASA	1.29E-24
Potri.002G022700.1.p	PtGASA7	XP_002300708.2	42	101	PF02704	GASA	1.38E-23
Potri.005G239000.2.p	PtGASA8	XP_002307719.1	39	98	PF02704	GASA	1.08E-29
Potri.005G239100.2.p	PtGASA9	KAH8509589.1	43	102	PF02704	GASA	1.31E-27

Table S3. Detailed information of GASA family genes identified in *A. thaliana, M. domestica, Populus tricocarpa, O. sativa*, **and *Prunus persica.***

Gene_name	Duplication mode
AtGASA1	tandem
AtGASA10	dispersed
AtGASA11	dispersed
AtGASA12	dispersed
AtGASA13	dispersed
AtGASA14	tandem
AtGASA15	tandem
AtGASA2	WGD or Segmental
AtGASA3	tandem
AtGASA4	tandem
AtGASA5	dispersed
AtGASA6	dispersed
AtGASA7	dispersed
AtGASA8	dispersed
AtGASA9	tandem
MdGASA1	tandem
MdGASA10	WGD or Segmental
MdGASA11	dispersed
MdGASA12	WGD or Segmental
MdGASA13	WGD or Segmental
MdGASA14	WGD or Segmental
MdGASA15	WGD or Segmental
MdGASA16	dispersed
MdGASA17	WGD or Segmental
MdGASA18	WGD or Segmental
MdGASA19	tandem
MdGASA2	WGD or Segmental

MdGASA20	WGD or Segmental
MdGASA21	tandem
MdGASA22	WGD or Segmental
MdGASA23	WGD or Segmental
MdGASA24	WGD or Segmental
MdGASA25	WGD or Segmental
MdGASA26	proximal
MdGASA27	dispersed
MdGASA28	WGD or Segmental
MdGASA3	WGD or Segmental
MdGASA4	WGD or Segmental
MdGASA5	tandem
MdGASA6	WGD or Segmental
MdGASA7	WGD or Segmental
MdGASA8	tandem
MdGASA9	proximal
OsGASA1	Dispersed
OsGASA11	Dispersed
OsGASA2	Dispersed
OsGASA3	Dispersed
OsGASA5	Dispersed
OsGASA6	Dispersed
OsGASA7	Dispersed
OsGASA8	Singleton
OsGASA9	Dispersed
PmGASA1	WGD or Segmental
PmGASA10	dispersed
PmGASA11	WGD or Segmental
PmGASA12	WGD or Segmental
PmGASA13	dispersed
PmGASA14	WGD or Segmental
PmGASA15	dispersed
PmGASA16	dispersed
PmGASA2	WGD or Segmental
PmGASA3	tandem
PmGASA4	tandem
PmGASA5	dispersed
PmGASA6	WGD or Segmental
PmGASA7	WGD or Segmental
PmGASA8	WGD or Segmental
PmGASA9	WGD or Segmental
PpGASA1	dispersed
PpGASA10	dispersed

PpGASA11	WGD or Segmental
PpGASA12	WGD or Segmental
PpGASA13	dispersed
PpGASA14	WGD or Segmental
PpGASA15	dispersed
PpGASA16	WGD or Segmental
PpGASA17	proximal
PpGASA2	WGD or Segmental
PpGASA3	WGD or Segmental
PpGASA4	tandem
PpGASA5	WGD or Segmental
PpGASA6	tandem
PpGASA7	WGD or Segmental
PpGASA8	WGD or Segmental
PpGASA9	WGD or Segmental
PtGASA1	Dispersed
PtGASA10	WGD or Segmental
PtGASA11	WGD or Segmental
PtGASA12	WGD or Segmental
PtGASA13	Dispersed
PtGASA14	WGD or Segmental
PtGASA15	Dispersed
PtGASA16	Dispersed
PtGASA17	WGD or Segmental
PtGASA18	Dispersed
PtGASA19	WGD or Segmental
PtGASA2	WGD or Segmental
PtGASA3	Dispersed
PtGASA4	WGD or Segmental
PtGASA5	WGD or Segmental
PtGASA6	Dispersed
PtGASA7	Dispersed
PtGASA8	WGD or Segmental
PtGASA9	Dispersed

Table S4. The cis-regulatory elements identified for *PmGASA* genes and their detailed information.

Gene	Site Name	Organism	Position	Strand	Sequence	Function
PmGASA1	ABRE	Hordeum vulgare	93	-	CGTACGTGCA	cis-acting element involved in the abscisic acid responsiveness
PmGASA1	ABRE	Arabidopsis thaliana	135	-	ACGTG	cis-acting element involved in the abscisic acid responsiveness
PmGASA1	ABRE	Arabidopsis thaliana	95	-	ACGTG	cis-acting element involved in the abscisic acid responsiveness
PmGASA1	ABRE	Arabidopsis thaliana	847	+	ACGTG	cis-acting element involved in the abscisic acid responsiveness
PmGASA1	AuxRR-core	Nicotiana tabacum	817	-	GGTCCAT	cis-acting regulatory element involved in auxin responsiveness
PmGASA1	G-box	Pisum sativum	135	+	CACGTT	cis-acting regulatory element involved in light responsiveness
PmGASA1	G-box	Pisum sativum	846	-	CACGTT	cis-acting regulatory element involved in light responsiveness
PmGASA1	G-box	Arabidopsis thaliana	95	-	TACGTG	cis-acting regulatory element involved in light responsiveness
PmGASA1	LTR	Hordeum vulgare	85	+	CCGAAA	cis-acting element involved in low-temperature responsiveness
PmGASA1	LTR	Hordeum vulgare	1143	+	CCGAAA	cis-acting element involved in low-temperature responsiveness
PmGASA1	MBS	Arabidopsis thaliana	174	+	CAACTG	MYB binding site involved in drought-inducibility
PmGASA1	MBS	Arabidopsis thaliana	1520	+	CAACTG	MYB binding site involved in drought-inducibility

PmGASA1	TATC-box	Oryza sativa	270	+	TATCCCA	cis-acting element involved in gibberellin-responsiveness
PmGASA1	TCA-element	Brassica oleracea	741	+	TCAGAAGAGG	cis-acting element involved in salicylic acid responsiveness
PmGASA1	TGA-element	Brassica oleracea	212	-	AACGAC	auxin-responsive element
PmGASA1	circadian	Lycopersicon esculentum	497	-	CAAAGATATC	cis-acting regulatory element involved in circadian control
PmGASA2	ABRE	Hordeum vulgare	690	+	CGCACGTGTC	cis-acting element involved in the abscisic acid responsiveness
PmGASA2	ACE	Petroselinum crispum	1402	-	CTAACGTATT	cis-acting element involved in light responsiveness
PmGASA2	CGTCA-motif	Hordeum vulgare	482	-	CGTCA	cis-acting regulatory element involved in the MeJA-responsiveness
PmGASA2	CGTCA-motif	Hordeum vulgare	282	-	CGTCA	cis-acting regulatory element involved in the MeJA-responsiveness
PmGASA2	CGTCA-motif	Hordeum vulgare	1100	-	CGTCA	cis-acting regulatory element involved in the MeJA-responsiveness
PmGASA2	G-box	Brassica oleracea	1559	+	TAACACGTAG	cis-acting regulatory element involved in light responsiveness
PmGASA2	GARE-motif	Brassica oleracea	1027	+	TCTGTTG	gibberellin-responsive element
PmGASA2	LTR	Hordeum vulgare	1283	+	CCGAAA	cis-acting element involved in low-temperature responsiveness
PmGASA2	MRE	Petroselinum crispum	152	-	AACCTAA	MYB binding site involved in light responsiveness

PmGASA2	TGA-element	Brassica oleracea	1033	-	AACGAC	auxin-responsive element
PmGASA2	TGA-element	Brassica oleracea	550	-	AACGAC	auxin-responsive element
PmGASA2	TGA-element	Brassica oleracea	253	-	AACGAC	auxin-responsive element
PmGASA2	TGACG-motif	Hordeum vulgare	1100	+	TGACG	cis-acting regulatory element involved in the MeJA-responsiveness
PmGASA2	TGACG-motif	Hordeum vulgare	282	+	TGACG	cis-acting regulatory element involved in the MeJA-responsiveness
PmGASA2	TGACG-motif	Hordeum vulgare	482	+	TGACG	cis-acting regulatory element involved in the MeJA-responsiveness
PmGASA3	ABRE	Arabidopsis thaliana	1222	-	ACGTG	cis-acting element involved in the abscisic acid responsiveness
PmGASA3	ABRE	Arabidopsis thaliana	981	-	ACGTG	cis-acting element involved in the abscisic acid responsiveness
PmGASA3	ABRE	Oryza sativa	1220	-	TACGTGTC	cis-acting element involved in the abscisic acid responsiveness
PmGASA3	ACE	Petroselinum crispum	1220	+	GACACGTATG	cis-acting element involved in light responsiveness
PmGASA3	G-box	Arabidopsis thaliana	981	-	TACGTG	cis-acting regulatory element involved in light responsiveness
PmGASA3	G-box	Arabidopsis thaliana	1222	-	TACGTG	cis-acting regulatory element involved in light responsiveness
PmGASA3	G-box	Zea mays	1982	+	CACGAC	cis-acting regulatory element involved in light responsiveness

PmGASA3	GARE-motif	Brassica oleracea	1989	-	TCTGTTG	gibberellin-responsive element
PmGASA3	LTR	Hordeum vulgare	1215	-	CCGAAA	cis-acting element involved in low-temperature responsiveness
PmGASA3	MRE	Petroselinum crispum	660	+	AACCTAA	MYB binding site involved in light responsiveness
PmGASA3	TCA-element	Nicotiana tabacum	1586	+	CCATCTTTTT	cis-acting element involved in salicylic acid responsiveness
PmGASA3	circadian	Lycopersicon esculentum	1512	-	CAAAGATATC	cis-acting regulatory element involved in circadian control
PmGASA3	circadian	Lycopersicon esculentum	1200	-	CAAAGATATC	cis-acting regulatory element involved in circadian control
PmGASA4	ABRE	Arabidopsis thaliana	1717	-	ACGTG	cis-acting element involved in the abscisic acid responsiveness
PmGASA4	ABRE	Oryza sativa	1715	-	TACGTGTC	cis-acting element involved in the abscisic acid responsiveness
PmGASA4	ABRE	Arabidopsis thaliana	136	+	ACGTG	cis-acting element involved in the abscisic acid responsiveness
PmGASA4	ABRE	Hordeum vulgare	159	+	CGTACGTGCA	cis-acting element involved in the abscisic acid responsiveness
PmGASA4	ACE	Petroselinum crispum	1715	+	GACACGTATG	cis-acting element involved in light responsiveness
PmGASA4	CGTCA-motif	Hordeum vulgare	1563	-	CGTCA	cis-acting regulatory element involved in the MeJA-responsiveness
PmGASA4	G-box	Arabidopsis thaliana	135	+	TACGTG	cis-acting regulatory element involved in light responsiveness

PmGASA4	G-box	Arabidopsis thaliana	1717	-	TACGTG	cis-acting regulatory element involved in light responsiveness
PmGASA4	TCA-element	Nicotiana tabacum	1332	+	CCATCTTTTT	cis-acting element involved in salicylic acid responsiveness
PmGASA4	TCA-element	Nicotiana tabacum	450	+	CCATCTTTTT	cis-acting element involved in salicylic acid responsiveness
PmGASA4	TGACG-motif	Hordeum vulgare	1563	+	TGACG	cis-acting regulatory element involved in the MeJA-responsiveness
PmGASA5	ABRE	Arabidopsis thaliana	395	-	ACGTG	cis-acting element involved in the abscisic acid responsiveness
PmGASA5	ABRE	Arabidopsis thaliana	294	+	ACGTG	cis-acting element involved in the abscisic acid responsiveness
PmGASA5	CGTCA-motif	Hordeum vulgare	1105	-	CGTCA	cis-acting regulatory element involved in the MeJA-responsiveness
PmGASA5	G-box	Pisum sativum	293	-	CACGTT	cis-acting regulatory element involved in light responsiveness
PmGASA5	G-box	Pisum sativum	395	+	CACGTT	cis-acting regulatory element involved in light responsiveness
PmGASA5	GT1-motif	Arabidopsis thaliana	399	-	GGTTAA	light responsive element
PmGASA5	LTR	Hordeum vulgare	23	-	CCGAAA	cis-acting element involved in low-temperature responsiveness
PmGASA5	LTR	Hordeum vulgare	510	+	CCGAAA	cis-acting element involved in low-temperature responsiveness
PmGASA5	LTR	Hordeum vulgare	1493	+	CCGAAA	cis-acting element involved in low-temperature responsiveness
PmGASA5	MBS	Arabidopsis thaliana	419	-	CAACTG	MYB binding site involved in drought-inducibility

PmGASA5	MBS	Arabidopsis thaliana	1806	-	CAACTG	MYB binding site involved in drought-inducibility
PmGASA5	MBSI	Petunia hybrida	1490	-	TTTTTACGGTTA	MYB binding site involved in flavonoid biosynthetic genes regulation
PmGASA5	P-box	Oryza sativa	1260	-	CCTTTTG	gibberellin-responsive element
PmGASA5	TCA-element	Nicotiana tabacum	1502	-	CCATCTTTTT	cis-acting element involved in salicylic acid responsiveness
PmGASA5	TGACG-motif	Hordeum vulgare	1105	+	TGACG	cis-acting regulatory element involved in the MeJA-responsiveness
PmGASA5	circadian	Lycopersicon esculentum	264	-	CAAAGATATC	cis-acting regulatory element involved in circadian control
PmGASA6	ABRE	Arabidopsis thaliana	1554	-	ACGTG	cis-acting element involved in the abscisic acid responsiveness
PmGASA6	ABRE	Arabidopsis thaliana	492	-	ACGTG	cis-acting element involved in the abscisic acid responsiveness
PmGASA6	AuxRR-core	Nicotiana tabacum	636	+	GGTCCAT	cis-acting regulatory element involved in auxin responsiveness
PmGASA6	CGTCA-motif	Hordeum vulgare	1464	-	CGTCA	cis-acting regulatory element involved in the MeJA-responsiveness
PmGASA6	CGTCA-motif	Hordeum vulgare	1970	+	CGTCA	cis-acting regulatory element involved in the MeJA-responsiveness
PmGASA6	G-box	Pisum sativum	492	+	CACGTT	cis-acting regulatory element involved in light responsiveness
PmGASA6	G-box	Arabidopsis thaliana	1554	-	TACGTG	cis-acting regulatory element involved in light responsiveness
PmGASA6	GT1-motif	Avena sativa	996	-	GGTTAAT	light responsive element
PmGASA6	GT1-motif	Arabidopsis thaliana	997	-	GGTTAA	light responsive element

PmGASA6	MBS	Arabidopsis thaliana	767	-	CAACTG	MYB binding site involved in drought-inducibility
PmGASA6	TGACG-motif	Hordeum vulgare	1464	+	TGACG	cis-acting regulatory element involved in the MeJA-responsiveness
PmGASA6	TGACG-motif	Hordeum vulgare	1970	-	TGACG	cis-acting regulatory element involved in the MeJA-responsiveness
PmGASA7	ABRE	Hordeum vulgare	1763	+	CGCACGTGTC	cis-acting element involved in the abscisic acid responsiveness
PmGASA7	ABRE	Arabidopsis thaliana	1754	+	ACGTG	cis-acting element involved in the abscisic acid responsiveness
PmGASA7	ABRE	Arabidopsis thaliana	1765	-	CACGTG	cis-acting element involved in the abscisic acid responsiveness
PmGASA7	ABRE	Arabidopsis thaliana	1766	+	ACGTG	cis-acting element involved in the abscisic acid responsiveness
PmGASA7	ACE	Petroselinum crispum	1763	-	GACACGTATG	cis-acting element involved in light responsiveness
PmGASA7	G-box	Pisum sativum	1765	-	CACGTG	cis-acting regulatory element involved in light responsiveness
PmGASA7	G-box	Pisum sativum	1753	-	CACGTT	cis-acting regulatory element involved in light responsiveness
PmGASA7	G-box	Brassica napus	1764	-	ACACGTGT	cis-acting regulatory element involved in light responsiveness
PmGASA7	G-box	Arabidopsis thaliana	1765	-	CACGTG	cis-acting regulatory element involved in light responsiveness
PmGASA7	HD-Zip 3	Arabidopsis thaliana	1558	-	GTAAT(G/C)ATTAC	protein binding site
PmGASA7	LTR	Hordeum vulgare	850	-	CCGAAA	cis-acting element involved in low-temperature responsiveness

PmGASA7	LTR	Hordeum vulgare	1175	+	CCGAAA	cis-acting element involved in low-temperature responsiveness
PmGASA7	TGA-element	Brassica oleracea	443	+	AACGAC	auxin-responsive element
PmGASA7	circadian	Lycopersicon esculentum	1546	-	CAAAGATATC	cis-acting regulatory element involved in circadian control
PmGASA8	ABRE	Arabidopsis thaliana	1556	-	ACGTG	cis-acting element involved in the abscisic acid responsiveness
PmGASA8	CGTCA-motif	Hordeum vulgare	478	-	CGTCA	cis-acting regulatory element involved in the MeJA-responsiveness
PmGASA8	CGTCA-motif	Hordeum vulgare	1878	-	CGTCA	cis-acting regulatory element involved in the MeJA-responsiveness
PmGASA8	CGTCA-motif	Hordeum vulgare	2	+	CGTCA	cis-acting regulatory element involved in the MeJA-responsiveness
PmGASA8	CGTCA-motif	Hordeum vulgare	55	+	CGTCA	cis-acting regulatory element involved in the MeJA-responsiveness
PmGASA8	G-box	Zea mays	1211	-	CACGAC	cis-acting regulatory element involved in light responsiveness
PmGASA8	G-box	Arabidopsis thaliana	1556	-	TACGTG	cis-acting regulatory element involved in light responsiveness
PmGASA8	GARE-motif	Brassica oleracea	500	+	TCTGTTG	gibberellin-responsive element
PmGASA8	GT1-motif	Arabidopsis thaliana	1033	-	GGTTAA	light responsive element
PmGASA8	GT1-motif	Arabidopsis thaliana	999	-	GGTTAA	light responsive element
PmGASA8	GT1-motif	Arabidopsis thaliana	1763	-	GGTTAA	light responsive element
PmGASA8	MBS	Arabidopsis thaliana	1548	+	CAACTG	MYB binding site involved in drought-inducibility

PmGASA8	MBS	Arabidopsis thaliana	1872	+	CAACTG	MYB binding site involved in drought-inducibility
PmGASA8	MBS	Arabidopsis thaliana	1509	-	CAACTG	MYB binding site involved in drought-inducibility
PmGASA8	TC-rich repeats	Nicotiana tabacum	1615	+	GTTTTCTTAC	cis-acting element involved in defense and stress responsiveness
PmGASA8	TC-rich repeats	Nicotiana tabacum	1568	+	ATTCTCTAAC	cis-acting element involved in defense and stress responsiveness
PmGASA8	TGACG-motif	Hordeum vulgare	478	+	TGACG	cis-acting regulatory element involved in the MeJA-responsiveness
PmGASA8	TGACG-motif	Hordeum vulgare	55	-	TGACG	cis-acting regulatory element involved in the MeJA-responsiveness
PmGASA8	TGACG-motif	Hordeum vulgare	1878	+	TGACG	cis-acting regulatory element involved in the MeJA-responsiveness
PmGASA8	TGACG-motif	Hordeum vulgare	2	-	TGACG	cis-acting regulatory element involved in the MeJA-responsiveness
PmGASA9	ABRE	Arabidopsis thaliana	594	+	ACGTG	cis-acting element involved in the abscisic acid responsiveness
PmGASA9	CGTCA-motif	Hordeum vulgare	246	-	CGTCA	cis-acting regulatory element involved in the MeJA-responsiveness
PmGASA9	G-box	Arabidopsis thaliana	593	+	TACGTG	cis-acting regulatory element involved in light responsiveness
PmGASA9	GT1-motif	Arabidopsis thaliana	814	-	GGTTAA	light responsive element
PmGASA9	GT1-motif	Arabidopsis thaliana	848	-	GGTTAA	light responsive element
PmGASA9	GT1-motif	Avena sativa	630	+	GGTTAAT	light responsive element
PmGASA9	LTR	Hordeum vulgare	430	-	CCGAAA	cis-acting element involved in low-temperature responsiveness
PmGASA9	MBS	Arabidopsis thaliana	1873	+	CAACTG	MYB binding site involved in drought-inducibility

PmGASA9	MBS	Arabidopsis thaliana	1394	+	CAACTG	MYB binding site involved in drought-inducibility
PmGASA9	MBS	Arabidopsis thaliana	341	-	CAACTG	MYB binding site involved in drought-inducibility
PmGASA9	MBS	Arabidopsis thaliana	739	-	CAACTG	MYB binding site involved in drought-inducibility
PmGASA9	TATC-box	Oryza sativa	1668	-	TATCCCA	cis-acting element involved in gibberellin-responsiveness
PmGASA9	TCA-element	Nicotiana tabacum	1661	-	CCATCTTTTT	cis-acting element involved in salicylic acid responsiveness
PmGASA9	TGACG-motif	Hordeum vulgare	246	+	TGACG	cis-acting regulatory element involved in the MeJA-responsiveness
PmGASA10	ABRE	Arabidopsis thaliana	1488	+	ACGTG	cis-acting element involved in the abscisic acid responsiveness
PmGASA10	ABRE	Arabidopsis thaliana	855	-	ACGTG	cis-acting element involved in the abscisic acid responsiveness
PmGASA10	ABRE	Arabidopsis thaliana	653	-	ACGTG	cis-acting element involved in the abscisic acid responsiveness
PmGASA10	ABRE	Arabidopsis thaliana	561	+	ACGTG	cis-acting element involved in the abscisic acid responsiveness
PmGASA10	AuxRR-core	Nicotiana tabacum	445	+	GGTCCAT	cis-acting regulatory element involved in auxin responsiveness
PmGASA10	CGTCA-motif	Hordeum vulgare	857	+	CGTCA	cis-acting regulatory element involved in the MeJA-responsiveness
PmGASA10	CGTCA-motif	Hordeum vulgare	1159	-	CGTCA	cis-acting regulatory element involved in the MeJA-responsiveness
PmGASA10	G-box	Arabidopsis thaliana	653	-	TACGTG	cis-acting regulatory element involved in light responsiveness

PmGASA10	G-box	Arabidopsis thaliana	560	+	TACGTG	cis-acting regulatory element involved in light responsiveness
PmGASA10	G-box	Zea mays	855	+	CACGTC	cis-acting regulatory element involved in light responsiveness
PmGASA10	G-box	Arabidopsis thaliana	1487	+	TACGTG	cis-acting regulatory element involved in light responsiveness
PmGASA10	GT1-motif	Arabidopsis thaliana	1916	-	GGTTAA	light responsive element
PmGASA10	MBS	Arabidopsis thaliana	1826	+	CAACTG	MYB binding site involved in drought-inducibility
PmGASA10	MRE	Petroselinum crispum	888	-	AACCTAA	MYB binding site involved in light responsiveness
PmGASA10	MRE	Petroselinum crispum	1255	+	AACCTAA	MYB binding site involved in light responsiveness
PmGASA10	TCA-element	Nicotiana tabacum	265	+	CCATCTTTTT	cis-acting element involved in salicylic acid responsiveness
PmGASA10	TCA-element	Nicotiana tabacum	1753	-	CCATCTTTTT	cis-acting element involved in salicylic acid responsiveness
PmGASA10	TCA-element	Nicotiana tabacum	1771	-	CCATCTTTTT	cis-acting element involved in salicylic acid responsiveness
PmGASA10	TCA-element	Nicotiana tabacum	1114	-	CCATCTTTTT	cis-acting element involved in salicylic acid responsiveness
PmGASA10	TGA-element	Brassica oleracea	1134	+	AACGAC	auxin-responsive element
PmGASA10	TGACG-motif	Hordeum vulgare	1159	+	TGACG	cis-acting regulatory element involved in the MeJA-responsiveness
PmGASA10	TGACG-motif	Hordeum vulgare	857	-	TGACG	cis-acting regulatory element involved in the MeJA-responsiveness

PmGASA11	ABRE	Arabidopsis thaliana	1574	+	ACGTG	cis-acting element involved in the abscisic acid responsiveness
PmGASA11	ABRE	Arabidopsis thaliana	1573	-	CACGTG	cis-acting element involved in the abscisic acid responsiveness
PmGASA11	ABRE	Hordeum vulgare	1571	-	GCAACGTGTC	cis-acting element involved in the abscisic acid responsiveness
PmGASA11	G-box	Pisum sativum	1573	-	CACGTG	cis-acting regulatory element involved in light responsiveness
PmGASA11	G-box	Brassica napus	1572	+	ACACGTGGC	cis-acting regulatory element involved in light responsiveness
PmGASA11	G-box	Zea mays	549	+	CACGAC	cis-acting regulatory element involved in light responsiveness
PmGASA11	G-box	Arabidopsis thaliana	1573	-	CACGTG	cis-acting regulatory element involved in light responsiveness
PmGASA11	MBS	Arabidopsis thaliana	289	+	CAACTG	MYB binding site involved in drought-inducibility
PmGASA11	MBS	Arabidopsis thaliana	80	-	CAACTG	MYB binding site involved in drought-inducibility
PmGASA11	MBS	Arabidopsis thaliana	320	-	CAACTG	MYB binding site involved in drought-inducibility
PmGASA11	MRE	Petroselinum crispum	1641	+	AACCTAA	MYB binding site involved in light responsiveness
PmGASA11	P-box	Oryza sativa	1558	-	CCTTTTG	gibberellin-responsive element
PmGASA11	TC-rich repeats	Nicotiana tabacum	792	+	ATTCTCTAAC	cis-acting element involved in defense and stress responsiveness
PmGASA11	TGA-element	Brassica oleracea	1213	-	AACGAC	auxin-responsive element
PmGASA12	ABRE	Arabidopsis thaliana	1589	-	ACGTG	cis-acting element involved in the abscisic acid responsiveness

PmGASA12	CGTCA-motif	Hordeum vulgare	1591	+	CGTCA	cis-acting regulatory element involved in the MeJA-responsiveness
PmGASA12	CGTCA-motif	Hordeum vulgare	734	+	CGTCA	cis-acting regulatory element involved in the MeJA-responsiveness
PmGASA12	G-box	Zea mays	1589	+	CACGTC	cis-acting regulatory element involved in light responsiveness
PmGASA12	MBS	Arabidopsis thaliana	1651	-	CAACTG	MYB binding site involved in drought-inducibility
PmGASA12	MRE	Petroselinum crispum	1508	-	AACCTAA	MYB binding site involved in light responsiveness
PmGASA12	TGA-element	Brassica oleracea	1360	-	AACGAC	auxin-responsive element
PmGASA12	TGA-element	Brassica oleracea	41	-	AACGAC	auxin-responsive element
PmGASA12	TGACG-motif	Hordeum vulgare	1591	-	TGACG	cis-acting regulatory element involved in the MeJA-responsiveness
PmGASA12	TGACG-motif	Hordeum vulgare	734	-	TGACG	cis-acting regulatory element involved in the MeJA-responsiveness
PmGASA12	circadian	Lycopersicon esculentum	631	+	CAAAGATATC	cis-acting regulatory element involved in circadian control
PmGASA12	circadian	Lycopersicon esculentum	1905	+	CAAAGATATC	cis-acting regulatory element involved in circadian control
PmGASA13	GT1-motif	Arabidopsis thaliana	1853	-	GGTTAA	light responsive element
PmGASA13	GT1-motif	Arabidopsis thaliana	1401	-	GGTTAA	light responsive element
PmGASA13	GT1-motif	Avena sativa	511	+	GGTTAAT	light responsive element
PmGASA13	LTR	Hordeum vulgare	729	+	CCGAAA	cis-acting element involved in low-temperature responsiveness

PmGASA13	MBS	Arabidopsis thaliana	889	+	CAACTG	MYB binding site involved in drought-inducibility
PmGASA13	MBS	Arabidopsis thaliana	769	+	CAACTG	MYB binding site involved in drought-inducibility
PmGASA13	P-box	Oryza sativa	439	+	CCTTTTG	gibberellin-responsive element
PmGASA13	TATC-box	Oryza sativa	1262	+	TATCCCA	cis-acting element involved in gibberellin-responsiveness
PmGASA13	TC-rich repeats	Nicotiana tabacum	1671	+	ATTCTCTAAC	cis-acting element involved in defense and stress responsiveness
PmGASA13	TC-rich repeats	Nicotiana tabacum	1815	+	ATTCTCTAAC	cis-acting element involved in defense and stress responsiveness
PmGASA13	TGA-element	Brassica oleracea	846	+	AACGAC	auxin-responsive element
PmGASA13	circadian	Lycopersicon esculentum	484	-	CAAAGATATC	cis-acting regulatory element involved in circadian control
PmGASA14	AuxRE	Glycine max	99	+	TGTCTCAATAAG	part of an auxin-responsive element
PmGASA14	CGTCA-motif	Hordeum vulgare	21	-	CGTCA	cis-acting regulatory element involved in the MeJA-responsiveness
PmGASA14	CGTCA-motif	Hordeum vulgare	1071	-	CGTCA	cis-acting regulatory element involved in the MeJA-responsiveness
PmGASA14	HD-Zip 3	Arabidopsis thaliana	1130	-	GTAAT(G/C)ATTAC	protein binding site
PmGASA14	TCA-element	Nicotiana tabacum	424	-	CCATCTTTTT	cis-acting element involved in salicylic acid responsiveness
PmGASA14	TGA-element	Brassica oleracea	1219	+	AACGAC	auxin-responsive element
PmGASA14	TGACG-motif	Hordeum vulgare	21	+	TGACG	cis-acting regulatory element involved in the MeJA-responsiveness

PmGASA14	TGACG-motif	Hordeum vulgare	1071	+	TGACG	cis-acting regulatory element involved in the MeJA-responsiveness
PmGASA15	ABRE	Arabidopsis thaliana	1725	+	ACGTG	cis-acting element involved in the abscisic acid responsiveness
PmGASA15	CCAAT-box	Hordeum vulgare	710	+	CAACGG	MYBHv1 binding site
PmGASA15	CGTCA-motif	Hordeum vulgare	1723	-	CGTCA	cis-acting regulatory element involved in the MeJA-responsiveness
PmGASA15	CGTCA-motif	Hordeum vulgare	969	-	CGTCA	cis-acting regulatory element involved in the MeJA-responsiveness
PmGASA15	G-box	Zea mays	1724	-	CACGTC	cis-acting regulatory element involved in light responsiveness
PmGASA15	GARE-motif	Brassica oleracea	1073	+	TCTGTTG	gibberellin-responsive element
PmGASA15	GT1-motif	Avena sativa	1268	+	GGTTAAT	light responsive element
PmGASA15	LTR	Hordeum vulgare	1105	-	CCGAAA	cis-acting element involved in low-temperature responsiveness
PmGASA15	LTR	Hordeum vulgare	1532	-	CCGAAA	cis-acting element involved in low-temperature responsiveness
PmGASA15	MBS	Arabidopsis thaliana	1867	+	CAACTG	MYB binding site involved in drought-inducibility
PmGASA15	MBS	Arabidopsis thaliana	1162	+	CAACTG	MYB binding site involved in drought-inducibility
PmGASA15	TCA-element	Nicotiana tabacum	1744	+	CCATCTTTTT	cis-acting element involved in salicylic acid responsiveness
PmGASA15	TGACG-motif	Hordeum vulgare	1723	+	TGACG	cis-acting regulatory element involved in the MeJA-responsiveness

PmGASA15	TGACG-motif	Hordeum vulgare	969	+	TGACG	cis-acting regulatory element involved in the MeJA-responsiveness
PmGASA16	CGTCA-motif	Hordeum vulgare	678	-	CGTCA	cis-acting regulatory element involved in the MeJA-responsiveness
PmGASA16	CGTCA-motif	Hordeum vulgare	227	-	CGTCA	cis-acting regulatory element involved in the MeJA-responsiveness
PmGASA16	CGTCA-motif	Hordeum vulgare	1387	-	CGTCA	cis-acting regulatory element involved in the MeJA-responsiveness
PmGASA16	G-box	Zea mays	1030	-	CACGAC	cis-acting regulatory element involved in light responsiveness
PmGASA16	GT1-motif	Avena sativa	92	+	GGTTAAT	light responsive element
PmGASA16	LTR	Hordeum vulgare	1958	-	CCGAAA	cis-acting element involved in low-temperature responsiveness
PmGASA16	MBS	Arabidopsis thaliana	1918	+	CAACTG	MYB binding site involved in drought-inducibility
PmGASA16	MBS	Arabidopsis thaliana	1191	-	CAACTG	MYB binding site involved in drought-inducibility
PmGASA16	MBSI	Petunia hybrida	345	-	aaaAaaC(G/C)GTTA	MYB binding site involved in flavonoid biosynthetic genes regulation
PmGASA16	TCA-element	Nicotiana tabacum	1153	+	CCATCTTTTT	cis-acting element involved in salicylic acid responsiveness
PmGASA16	TGA-element	Brassica oleracea	1808	+	AACGAC	auxin-responsive element
PmGASA16	TGA-element	Brassica oleracea	2	-	AACGAC	auxin-responsive element
PmGASA16	TGACG-motif	Hordeum vulgare	1387	+	TGACG	cis-acting regulatory element involved in the MeJA-responsiveness

PmGASA16	TGACG-motif	Hordeum vulgare	227	+	TGACG	cis-acting regulatory element involved in the MeJA-responsiveness
PmGASA16	TGACG-motif	Hordeum vulgare	678	+	TGACG	cis-acting regulatory element involved in the MeJA-responsiveness
